# Temporal Partitioning of Carotenoid, Flavonoid and Anthocyanin Biosynthesis Underlies the Ontogenetic Petal Color Transition in *Weigela japonica*

**DOI:** 10.3390/metabo16070511

**Published:** 2026-07-22

**Authors:** Mei Zhang, Siyang Duan, Ji Zhang, Riwen Fei, Xiuting Zhao, Changbo Ji, Li Liu

**Affiliations:** 1 College of Science, Liaodong University, Dandong 118003, China; zhangmei@liaodongu.edu.cn; 2 College of Agriculture, Liaodong University, Dandong 118003, China; 923014@liaodong.edu.cn (R.F.); 924080@liaodongu.edu.cn (X.Z.); jizhangbo@liaodongu.edu.cn (C.J.); 3 Taizhixing Park Management Co., Ltd., Tianjin 300457, China; saberzj940501@gmail.com

**Keywords:** *Weigela japonica*, flower color, metabolomics, transcriptomics

## Abstract

**Background:** Ontogenetic flower color change is a prevalent adaptive phenomenon in plants, yet the temporal orchestration of multiple pigment pathways during rapid developmental transitions remains poorly understood. This study aims to elucidate the metabolic and transcriptional basis of the petal basal marking color transition in Weigela japonica from yellow through yellow-orange to purple-red within a 4-day blooming period. **Methods:** Metabolomic analysis of flavonoids, anthocyanins, and carotenoids was conducted using UPLC-MS/MS across four developmental stages (S0–S3). Differentially accumulated metabolites were screened based on VIP > 1, |Log_2_FC| ≥ 1, and *p* < 0.05. Transcriptome sequencing was performed using the NovaSeq 6000 platform, and differentially expressed genes were identified using DESeq2. Weighted gene co-expression network analysis (WGCNA) was employed to identify hub genes associated with pigment accumulation. Key structural genes were validated by qRT-PCR. **Results:** Metabolomics revealed distinct stage-specific pigment accumulation: carotenoids peaked during the yellow-orange stage (S2), while anthocyanins and flavonoids surged in the final purple-red stage (S3). KEGG enrichment indicated that carotenoid metabolism operates as a discrete module, anthocyanin biosynthesis as a terminal-specific route, and flavonoid metabolism as an intermediary hub bridging the two pathways. Transcriptome analysis identified stage-specific gene expression patterns: PAL and C4H were active at S0–S1, CHS/CHI/F3H peaked at S1-S2, and DFR/ANS were maximally expressed at S2–S3. Carotenoid genes (PSY, LCY, ZDS) showed synchronous expression during the yellow-orange phase. WGCNA identified MYB44, bHLH92, and ERF3 as hub genes strongly correlated with the S3 anthocyanin surge. **Conclusions:** These findings suggest a sequential activation model in *W. japonica*, in which temporally partitioned pigment metabolism and stepwise transcriptional regulation converge to orchestrate rapid ontogenetic color change. The study provides a framework for understanding developmental color transitions in ornamental plants, though causal regulatory relationships remain to be functionally validated.

## 1. Introduction

Among horticultural plants, flower color ranks as one of the most appealing ornamental traits [1]. On Earth, the majority of ornamental plant species exhibit uniform and pure flower coloration. A small subset, however, shows dynamic color variation in petal markings during development and anthesis. *Weigela japonica* offers a typical example. The color differentiation of markings predominantly occurs at the basal region of petals, and the altered color phenotype can be maintained for a certain duration after color transformation [2]. This ontogenetic flower color change during flower organ development and blooming is generally considered an adaptive mechanism that enhances the long-distance attraction of pollinators and reduces pollinator residence time on individual inflorescences [3]. From an evolutionary perspective, dynamic flower color variation improves flower attractiveness to pollinators, thereby facilitating plant pollen dispersal and reproductive success [4]. Meanwhile, this unique color-shifting characteristic significantly elevates ornamental value. It has attracted extensive attention from botanists and horticultural enthusiasts and has become a core breeding target for the innovation and improvement of ornamental plant cultivars [5].

Flower color diversity mainly stems from differential accumulation of endogenous pigments in plant tissues [6]. Four major classes of natural plant pigments, which include chlorophylls, carotenoids, anthocyanins, and flavonoids, have been recognized as the principal determinants of flower coloration [7,8,9]. Chlorophylls endow plants with green hues, while carotenoids typically generate yellow, orange and red color tones, and anthocyanins mediate red, purple and blue coloration whose specific shades are modulated by vacuolar pH and co-pigmentation effects [10]. Flavonoids are water-soluble phenylpropanoid-derived compounds bearing a characteristic C6-C3-C6 carbon backbone. They display broad chromatic versatility, contributing to orange, red, purple, and blue flower colors [11]. Over 9000 structurally distinct flavonoid compounds have been identified to date. This makes flavonoids one of the most diverse and functionally complex families of plant secondary metabolites [12,13]. These compounds are divided into six major subclasses that cover anthocyanins, flavones, flavonols, flavanones, isoflavones and chalcones. Flavonoids occur widely across various plant organs. They perform multiple well-documented biological functions, including antioxidant activity, anti-inflammatory effects, free radical scavenging, and cardioprotection. Because of this, they stand out as core functional phytochemicals and natural antioxidants in plants [14,15].

Anthocyanins are water-soluble pigments that contribute to red, purple, and blue flower colors [16]. Differences in aglycone backbone structures divide anthocyanins mainly into six common types: cyanidin, pelargonidin, petunidin, delphinidin, malvidin, and peonidin [17,18,19]. A series of crucial structural enzymes catalyze the anthocyanin biosynthetic pathway in sequence. These include phenylalanine ammonia-lyase (PAL), cinnamate 4-hydroxylase (C4H), 4-coumarate:CoA ligase (4CL), chalcone synthase (CHS), chalcone isomerase (CHI), flavanone 3-hydroxylase (F3H), flavonoid 3′-hydroxylase (F3’H), dihydroflavonol 4-reductase (DFR) and anthocyanin synthase (ANS) [20,21,22]. The transcriptional expression of these structural genes directly regulates the synthesis and accumulation of anthocyanins, which are coordinately regulated by a conserved MYB-bHLH-WDR (MBW) transcriptional complex in plants [23].

During flower development, certain transcription factors show spatiotemporal expression in specific petal regions [24]. This gives rise to gradual flower color changes as blooming progresses. Anthocyanin synthesis, meanwhile, is highly dynamic [25]. At different stages of flower development, genes linked to various pigments are expressed differently [26]. In early blooming, for instance, chlorophyll- or carotenoid-related genes tend to dominate. In mid or final stages, genes in the anthocyanin metabolic pathway start showing specific high-level expression [27]. This temporal pattern of gene expression directly leads to substantial changes in pigment content and color intensity throughout different phases of petal opening [28]. In addition to direct activation by transcription factors, epigenetic modifications, such as DNA methylation, also exert a profound influence on the development of variegated patterns. Studies show that in some plant petals, promoter methylation levels of key anthocyanin biosynthesis genes vary significantly across flowering stages [29,30]. High levels of DNA methylation can silence these critical enzyme genes, ensuring that anthocyanins accumulate specifically over time during the opening process. This accumulation forms distinct patterns that become increasingly apparent as the flower develops [31,32].

Integrated transcriptomic and metabolomic analysis has become a powerful and prevalent approach for analyzing the molecular regulatory mechanisms during flower color formation and variation in ornamental plants. Transcriptomic sequencing allows systematic identification of differentially expressed genes (DEGs) involved in pigment biosynthesis, transcriptional regulation, and flower development. Metabolomics, on the other hand, can accurately quantify dynamic accumulation of pigment compounds and intermediate metabolites during flower maturation [33,34,35]. Combining these two omics strategies builds a strong link between gene expression patterns and metabolic phenotypic changes. It effectively bridges the gap between genetic regulation and downstream metabolite accumulation. Numerous recent studies have adopted this integrated strategy to elucidate the core regulatory networks of flavonoid and anthocyanin biosynthesis, identifying transcription factors, key genes, and differentially accumulated metabolites that dominate flower color differentiation and ontogenetic color transformation. Compared with single-omics analysis, multi-omics integration provides more comprehensive and reliable evidence for revealing the complex molecular mechanisms of plant color traits, which lays a solid theoretical foundation for the molecular mechanism exploration and precision breeding of color-variation ornamental plant species [36,37,38]. Previous studies have reported that differential accumulation of malvidin 3,5-diglucoside contributes to flower color diversity [39]. Further transcriptomics analysis of differentially expressed related genes can shed more light on this [40]. In addition, higher levels of 7 anthocyanins explained deeper pigmentation in miniature roses. This aligns with elevated expression levels of 13 anthocyanin biosynthesis genes found through integrated metabolomics and transcriptomics analysis [41]. Overall, these findings will provide insights for breeding new ornamental plant varieties with novel flower colors.

*W. japonica*, a deciduous shrub in the Caprifoliaceae family, holds considerable ornamental value due to its distinctive ontogenetic petal color variation across the flowering period. During its 4-day blossoming process, the petal basal markings of *W. japonica* exhibit a regular sequential color transition from pure yellow to yellow-orange and ultimately to purplish red. Specifically, the pure yellow and yellow-orange phases each last for approximately 1 day, whereas the purplish red phase lasts for 2 days. Interestingly, flowers lose most male and female reproductive capacity after the color transformation completes. This unique temporal color pattern is thought to be closely linked to pollen tube growth progression [2]. Despite the striking phenotypic characteristics and high ornamental value of *W. japonica* color change, the metabolic basis and genetic regulatory mechanisms driving its dynamic flower color transformation are still unknown. In this study, flower samples of *W. japonica* at different developmental stages with distinct color phenotypes were collected as research materials. Through integrated transcriptomic and metabolomic profiling, this study screened differentially accumulated metabolites and pinpointed key genes for stage-specific flower color formation. The findings of this work will provide novel insights into the molecular regulatory mechanism of ontogenetic flower color variation in *W. japonica*. They will also provide a theoretical reference for molecular breeding of color-variable ornamental plants.

## 2. Materials and Methods

### 2.1. Plant Material

**Figure 1:**
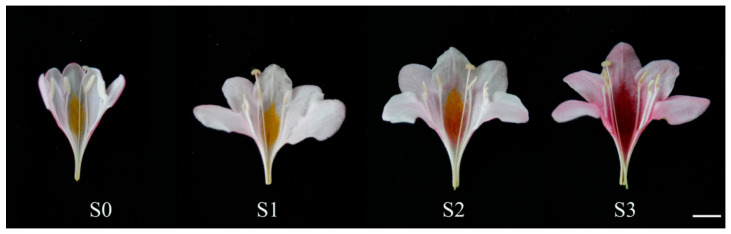
Flowers of *W. japonica* and phenotypic color variation at different flowering stages. The color within the flower alters as it blossoms over time. S0 represents the initial stage of flower opening, S1 denotes one day after flowering, S2 indicates two days after flowering, and S3 signifies three to four days after flowering. The bar is 0.5 cm.

Plant materials were collected from healthy *Weigela japonica* shrubs grown on the campus of Liaodong University, Dandong, Liaoning Province, China. Petal samples covering four continuous flower developmental stages (S0–S3) were harvested in May 2025. The grouping standard is primarily based on fixed post-anthesis flowering time, with synchronized petal basal marking color as an auxiliary phenotypic reference: S0 represents newly bloomed flowers; S1 indicates flowers 1 day after anthesis; S2 denotes flowers 2 days after anthesis; S3 covers flowers at 3–4 days post-anthesis. Under consistent cultivation and light conditions, flowers at the same developmental time point exhibit highly uniform and consistent basal marking color without obvious individual variation, which ensures the repeatability of sample grouping. All freshly collected petals were wrapped with tin foil, snap-frozen in liquid nitrogen immediately, and stored at −80 °C for subsequent metabolomic and transcriptomic analysis. Phenotypic photographs of petals at each stage are presented in Figure 1 to intuitively display the stable color characteristics corresponding to each developmental time gradient.

### 2.2. Metabolic Extraction and Analysis

Petal samples of *W. japonica* were divided into 4 groups, namely S0, S1, S2, and S3, with 3 biological replicates in each group. This led to a total of 12 specimens being used for the simultaneous quantification of anthocyanins, carotenoids, and flavonoids via ultra-performance liquid chromatography coupled with tandem mass spectrometry (UPLC-MS/MS). All plant samples were freeze-dried and then homogenized into powder before metabolite extraction. For anthocyanin extraction, 50 mg of sample was mixed with 500 μL of 50% aqueous methanol (Macklin, Shanghai, China) containing 0.1% hydrochloric acid (Macklin, Shanghai, China). Subsequently, the mixed solution was subjected to successive vortexing and ultrasonication. Then, the mixed solution was centrifuged at 12,000 r/min at 4 °C. The supernatants obtained from two parallel extractions were combined and then filtered through a 0.22 μm membrane. For carotenoid extraction, 50 mg of sample powder was extracted using a mixed solution of hexane (Xilong, Shenzhen, China), acetone (Xilong, Shenzhen, China), and ethanol (Xilong, Shenzhen, China) supplemented with 0.01% butylated hydroxytoluene (BHT) (Macklin, Shanghai, China). The pooled supernatants were then concentrated and reconstituted with dichloromethane (Xilong, Shenzhen, China) prior to filtration. For flavonoid detection, 20 mg of sample powder was extracted using 500 μL of a 70% methanol solution containing an internal standard. Subsequently, the mixed solution was ultrasonicated, centrifuged, and filtered accordingly. Chromatographic separation was conducted on an ExionLC™ AD UPLC system equipped with an ACQUITY UPLC HSS T3 C18 column (1.8 um, 2.1 mm × 100 mm; Waters, Milford, MA, USA). The mobile phase consisted of solvent A (0.1% formic acid in water, *v*/*v*) and solvent B (acetonitrile). The gradient elution program was set as follows: 0–1 min, 5% B; 1–9 min, 5–50% B; 9–12 min, 50–95% B; 12–13.5 min, 95% B; 13.5–14 min, 95–5% B; 14–15 min, 5% B. The flow rate was 0.35 mL/min, the column temperature was maintained at 40 degC, and the injection volume was 2 uL. Meanwhile, mass spectrometry detection was executed on a SCIEX QTRAP 6500+ instrument (SCIEX, Shanghai, China) equipped with either an electrospray ionization (ESI) or an atmospheric pressure chemical ionization (APCI) source under optimized parameters. The multiple reaction monitoring (MRM) mode was employed for the targeted scanning of all candidate metabolites. A series of mixed standard solutions with gradient concentrations were prepared to establish standard curves for absolute quantification. Subsequently, metabolite contents were calculated based on peak areas, standard curve equations, and corresponding conversion formulas. Quality control (QC) samples were inserted at regular intervals during the detection process. Total ion current (TIC) overlay plots and the coefficient of variation (CV) distribution of QC samples were utilized to assess instrument stability and data reproducibility. Raw mass spectral data were processed using Analyst (v1.7.1)and MultiQuant (v3.0.3) software for peak identification and integration. All quantitative data were normalized using unit variance scaling (Z-score) for subsequent bioinformatic analyses.

Hierarchical cluster analysis (HCA) was carried out to profile metabolite accumulation patterns and overall metabolic differences among samples. Orthogonal partial least squares-discriminant analysis (OPLS-DA) was conducted following mean centering to distinguish inter-group variations, and permutation tests were employed to validate the reliability of the model. Differentially accumulated metabolites were screened by combining the variable importance in projection (VIP > 1) from OPLS-DA, fold change (FC ≥ 2 or FC ≤ 0.5) for pairwise comparisons, and one-way ANOVA (*p* < 0.05) for multi-group comparisons. The identified differentially accumulated metabolites were further annotated against the Kyoto Encyclopedia of Genes and Genomes (KEGG) database, and pathway enrichment analysis based on the hypergeometric test was implemented to characterize the functional pathways associated with the differentially accumulated metabolites.

### 2.3. RNA Extraction, Sequencing, and Functional Annotation of Transcripts

Total RNA was isolated from all plant samples using a combined CTAB and pBIOZOL reagent method. The purity, concentration and integrity of the extracted RNA were rigorously assessed using a Qubit 4.0 fluorometer (Thermo Fisher Scientific, Waltham, MA, USA), a SpectraMax M2 microplate reader (Molecular Devices, San Jose, CA, USA) and a Qsep400 nucleic acid analysis (BiOptic, New Taipei City, Taiwan) system to ensure all samples met the criteria for library construction. RNA sequencing was performed on the Illumina NovaSeq 6000 platform (Illumina, San Diego, CA, USA) using 150 bp paired-end sequencing. Each sample generated 44.13–67.54 million clean reads (6.62–10.13 Gb), with Q20 > 99% and Q30 > 96.9%. Raw sequencing reads were filtered by employing the fastp software (v0.24.0) to eliminate adapter-contaminated reads, reads containing more than 10% ambiguous bases (N), and reads with over 50% low-quality bases (Q ≤ 20), thereby obtaining high-quality clean reads for subsequent analysis. De novo transcriptome assembly was carried out using the Trinity software (v2.15.2), and redundant transcripts were clustered and removed with Corset to produce unigenes. Using TransDecoder to predict the coding sequences (CDSs) of assembled transcripts. Clean reads were mapped to the assembled transcriptome using HISAT2 (v2.2.1). The mapping rate ranged from 86.69% to 88.06% across samples (mean 87.50%). The unique mapping rate ranged from 27.59% to 30.21% (mean 28.91%), and the multiple mapping rate ranged from 57.32% to 60.47% (mean 58.59%).

Gene expression levels were precisely quantified by RSEM software (v1.3.3), and the expression value of each unigene was accurately calculated as fragments per kilobase of transcript per million mapped reads (FPKM). Differential expression analysis between sample groups was rigorously conducted using DESeq2 for samples with biological replicates. The Benjamini–Hochberg method was applied to appropriately correct the *p*-values for multiple testing. Genes with |log_2_(fold change)| ≥ 1 and false discovery rate (FDR) < 0.05 were clearly defined as DEGs. Moreover, GO and KEGG pathway enrichment analyses of DEGs were effectively implemented based on the hypergeometric distribution test to explore the biological functions and metabolic pathways associated with DEGs.

### 2.4. Gene-Metabolite Correlation Analysis

Pearson correlation coefficients were calculated between the expression levels (FPKM) of key structural genes and the accumulation levels of 58 differentially accumulated metabolites across all four developmental stages (S0–S3) using the corrplot package in R (v4.3.0). Gene-metabolite pairs with |r| ≥ 0.8 and *p* < 0.05 were defined as significantly correlated. The correlation matrix was visualized using the pheatmap R package (v1.0.13; Kolde 2025), with hierarchical clustering (Euclidean distance, complete linkage) applied to both rows and columns. Significant gene-metabolite pairs were identified by filtering the pre-computed gene-to-metabolite correlation table generated during the metabolome-transcriptome joint analysis (MetWare, Wuhan, China).

### 2.5. qRT-PCR Analysis

qRT-PCR was performed using the 2X HyperMB Universal SYBR Green qPCR Master Mix (BBI). The reaction protocol was as follows: pre-denaturation at 95 °C for 30 s. The amplification cycles, consisting of 95 °C for 5 s and 60 °C for 30 s, were repeated 40 times. Melting curve analysis was then conducted at 95 °C for 15 s, 60 °C for 1 min, and 95 °C for 15 s. Each experiment included three biological replicates and three technical replicates. All primers used in the current study are presented in Table S1, and Actin was utilized to standardize the gene expression level. Relative gene expression levels were calculated using the 2^−∆∆Ct^ method. Data were analyzed for standard deviation and statistical significance using SPSS (v27.0) software, and bar graphs were generated with GraphPad Prism 11.0.

### 2.6. Statistical Analysis

All quantitative data in this study were obtained with three independent biological replicates. Prior to one-way analysis of variance (ANOVA), the Shapiro–Wilk test was performed to verify the normal distribution of residuals, and Levene’s test was used to assess homogeneity of variance. Only datasets satisfying normal distribution and homogeneous variance were subjected to subsequent one-way ANOVA, and the corresponding F-statistic values were recorded for all ANOVA analyses. When significant differences (*p* < 0.05) were detected among groups, Duncan’s multiple range test was applied for post hoc multiple comparisons. All statistical calculations were conducted using IBM SPSS Statistics 27. Error bars in all figures represent the standard deviation (SD) of biological replicates.

## 3. Results

### 3.1. Stage-Specific Metabolite Profiles Reveal Temporal Partitioning of Pigment Accumulation

**Figure 2:**
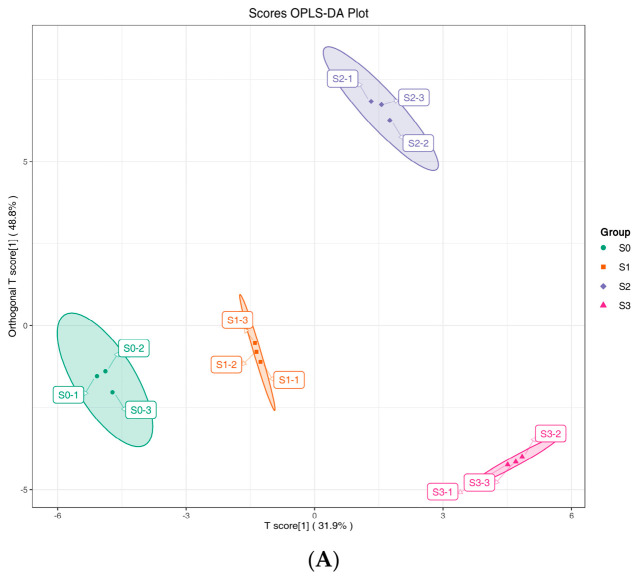

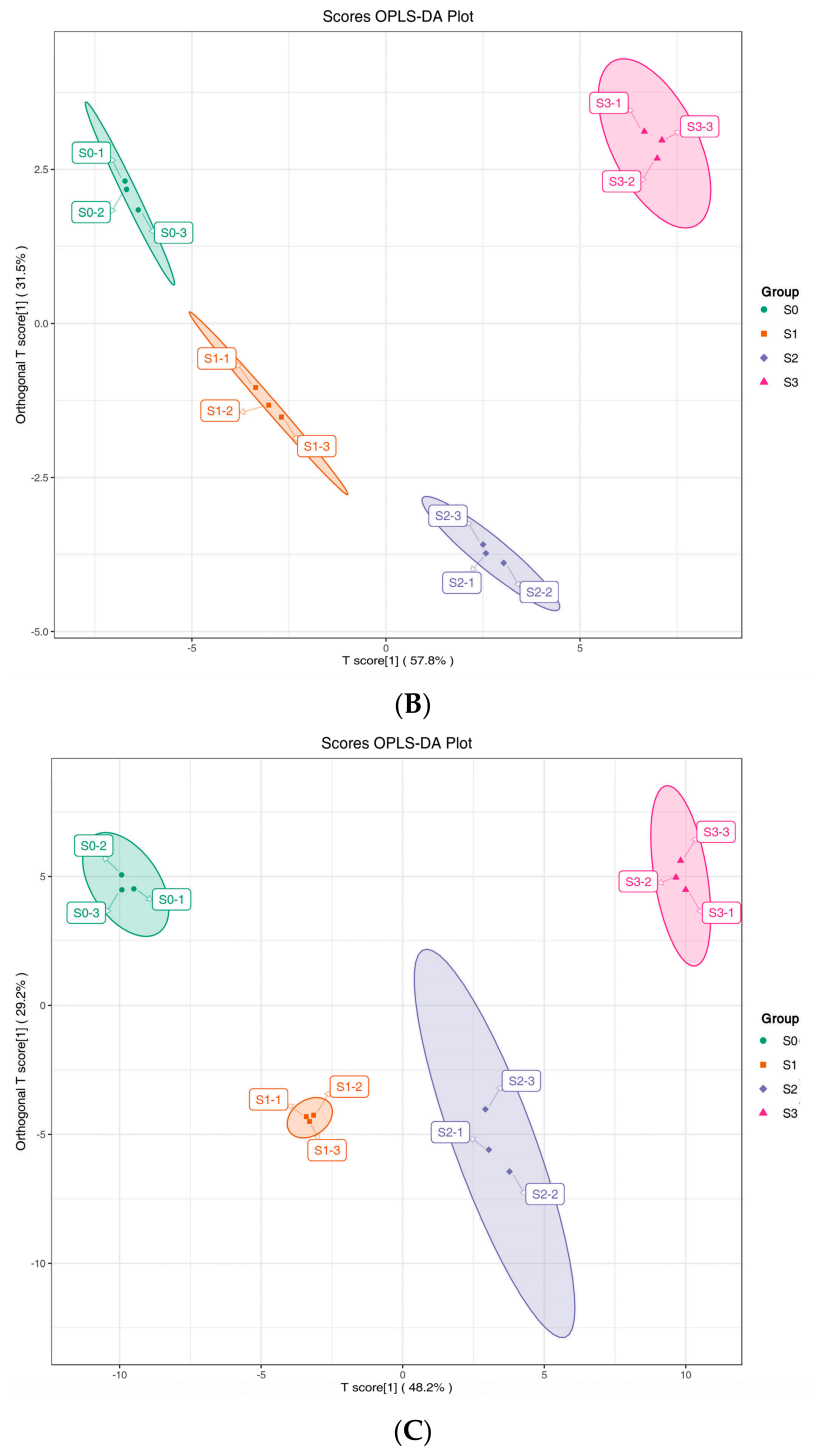
OPLS-DA analysis was conducted on carotenoids, anthocyanins, and flavonoids. (**A**) OPLS-DA analysis of carotenoids. (**B**) OPLS-DA analysis of anthocyanins. (**C**) OPLS-DA analysis of flavonoids. The predictive principal component (*x*-axis) separates the groups, whereas the orthogonal principal component (*y*-axis) captures intra-group variability. The attached percentages show the variance contribution of each component. Individual samples appear as separate points in the plot, with color-coding used to distinguish group membership.

UPLC-MS/MS metabolomics was applied to analyze flavonoids, anthocyanins, and carotenoids in 12 petal samples (S0–S3, 3 biological replicates per group) in order to uncover the metabolic basis of petal coloration in *W. japonica*. The TIC chromatograms of QC samples exhibited excellent overlap across three repeated injections, which indicated a high level of instrument stability (Figure S1A–C). Empirical cumulative distribution function (ECDF) analysis demonstrated that over 80% of the detected metabolites had CV below 0.2 across all 3 groups, thus meeting the reliability criteria for metabolomics data (Figure S2A–C). In total, 311 flavonoid metabolites, 49 anthocyanin metabolites, and 72 carotenoid metabolites were successfully annotated, including flavones, flavonols, flavanones, catechins, anthocyanin glycosides, proanthocyanidins, carotenes, lutein, and their esterified derivatives. Among all annotated metabolites, flavonoids formed the largest class by a considerable margin. Their overwhelming predominance implicates flavonoid metabolism as a key process underlying both the establishment and the shift in petal color in *W. japonica*. OPLS-DA clearly distinguished the 4 sample groups, with biological replicates closely clustered, which confirmed a high degree of reproducibility among samples and significant differences in metabolic profiles across developmental stages (Figure 2). The results clearly demonstrate that higher levels of carotenoid-related metabolites were detected in stage S2, whereas elevated concentrations of anthocyanins and flavonoids were observed in stage S3. This suggests that the color change in *W. japonica* is closely correlated with the content of these metabolites, and their levels may fluctuate accordingly as the flower color changes.

**Figure 3:**
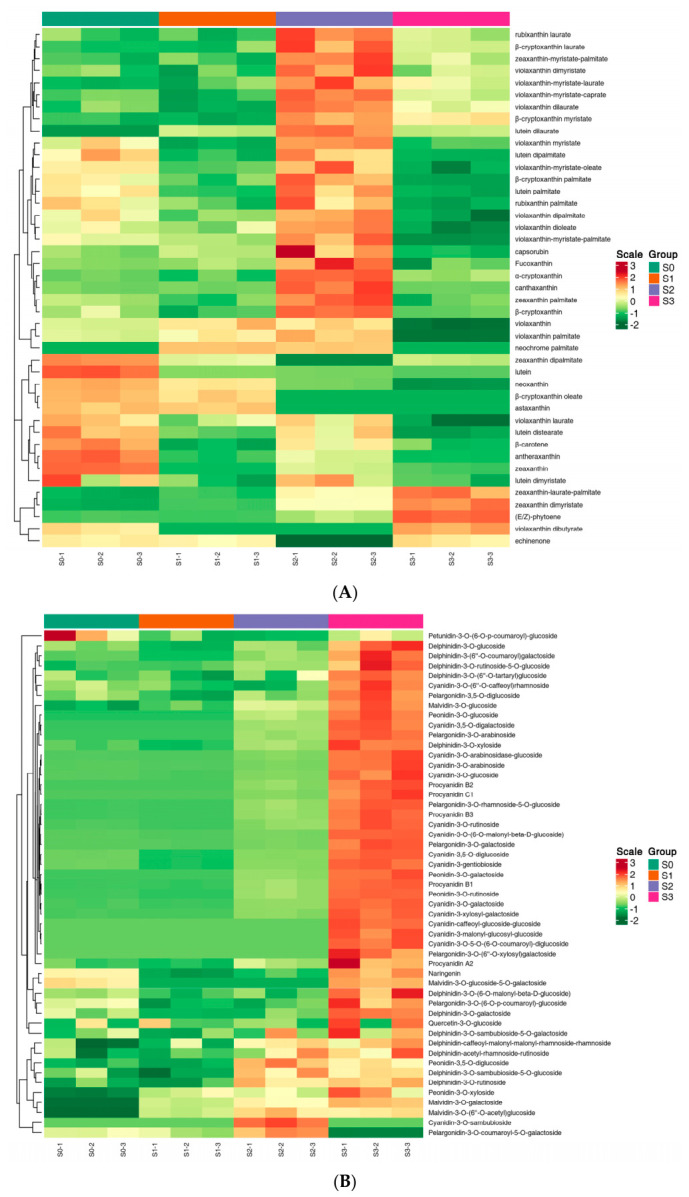

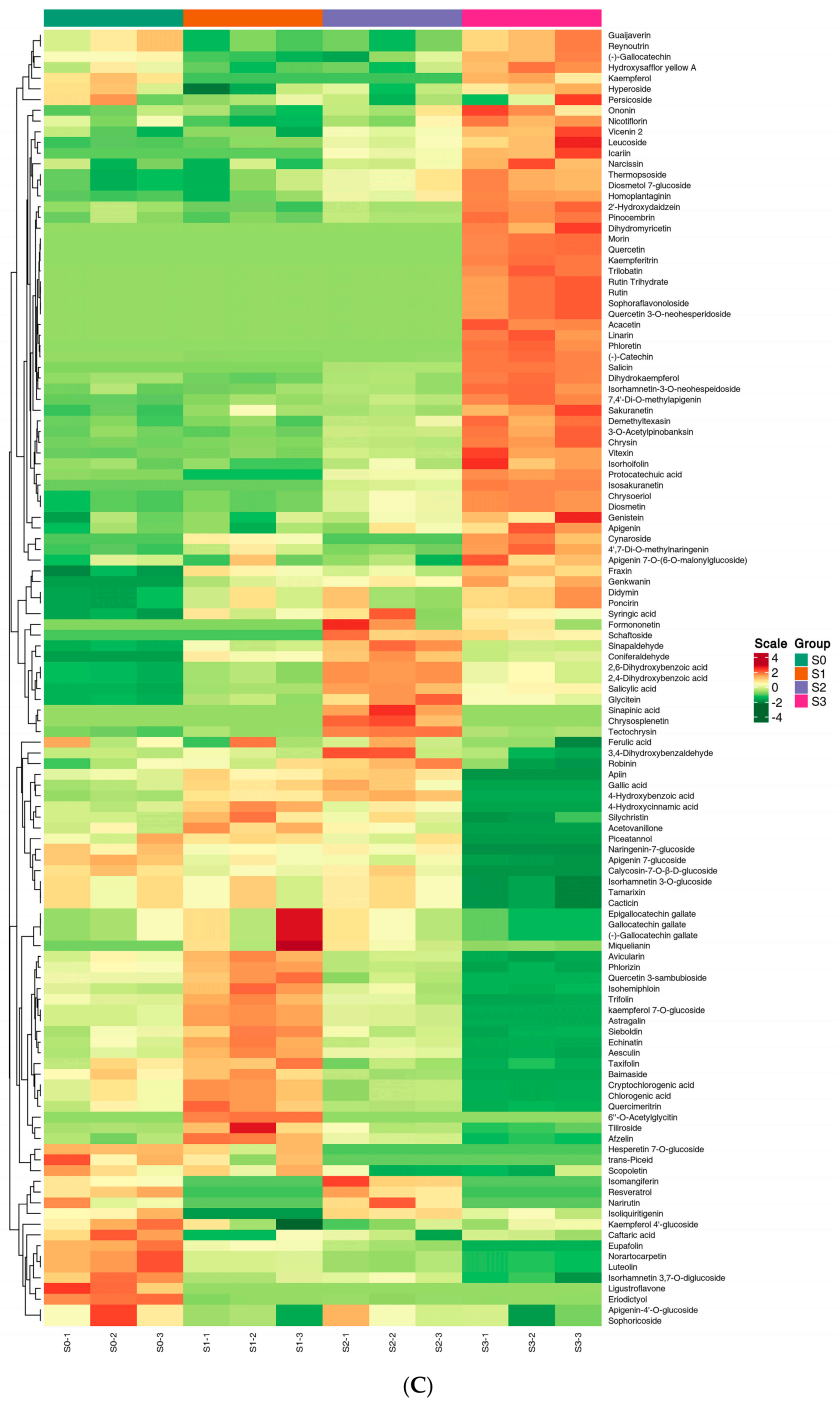
Cluster analysis of carotenoids, anthocyanins, and flavonoids during different flowering stages. (**A**) Heat map of carotenoids at different stages. (**B**) Heat map of anthocyanins at different stages. (**C**) Heat map of flavonoids at different stages. Different colors represent distinct values acquired after standardization according to varying concentrations (red denotes high concentration, while green denotes low concentration).

HCA heat maps further corroborated the stage-specific accumulation patterns of metabolites (Figure 3). Based on the results, it can be observed that the majority of carotenoid metabolites were relatively abundant during the S2 stage. Conversely, a substantial quantity of anthocyanins and flavonoid metabolites were detected in the S3 stage. This further validates the significant roles that these metabolites play at different flowering stages in *W. japonica*.

The differentially accumulated metabolites were analyzed in terms of the total metabolite accumulation across the four developmental stages. Normality testing (Shapiro–Wilk) and homogeneity of variance (Levene’s test) confirmed that all metabolite data met parametric assumptions, permitting the use of one-way ANOVA with Duncan’s multiple range test. The total metabolite accumulation of carotenoids peaked at the S2 stage and was significantly higher than that of the other three stages (F3,8 = 155.29, *p* < 0.001). The S0 stage and S1 stage exhibited a relatively similar accumulation level. The metabolite accumulation in the S2 stage showed a significant rise compared to the S0 and S1 stages, and subsequently dropped to a markedly low level in the S3 stage. The total metabolite accumulation of anthocyanins was notably lower in the S0 and S1 stages, with a significant rise observed in the S2 stage (F3,8 = 868.67, *p* < 0.001). The metabolite accumulation in the S3 stage experienced a significant increase compared to the previous three stages, reaching a markedly high level. The increase in the S3 stage was particularly dramatic, with a 4.91-fold change relative to S2. The total metabolite accumulation of flavonoids was highest in the S0 and S1 stages, with a significant decline beginning in the S2 stage (F3,8 = 155.25, *p* < 0.001). The metabolite accumulation in the S3 stage dropped to a markedly low level, reaching only 27.8% of the S0 stage. The S0 and S1 stages maintained similar accumulation levels, while the S2 and S3 stages showed progressive decreases. The ANOVA summary for total metabolite accumulation is provided in Table S1.

### 3.2. Differential Accumulation Highlights a Metabolic Shift from Carotenoid to Flavonoid-Anthocyanin Biosynthesis

**Figure 4:**
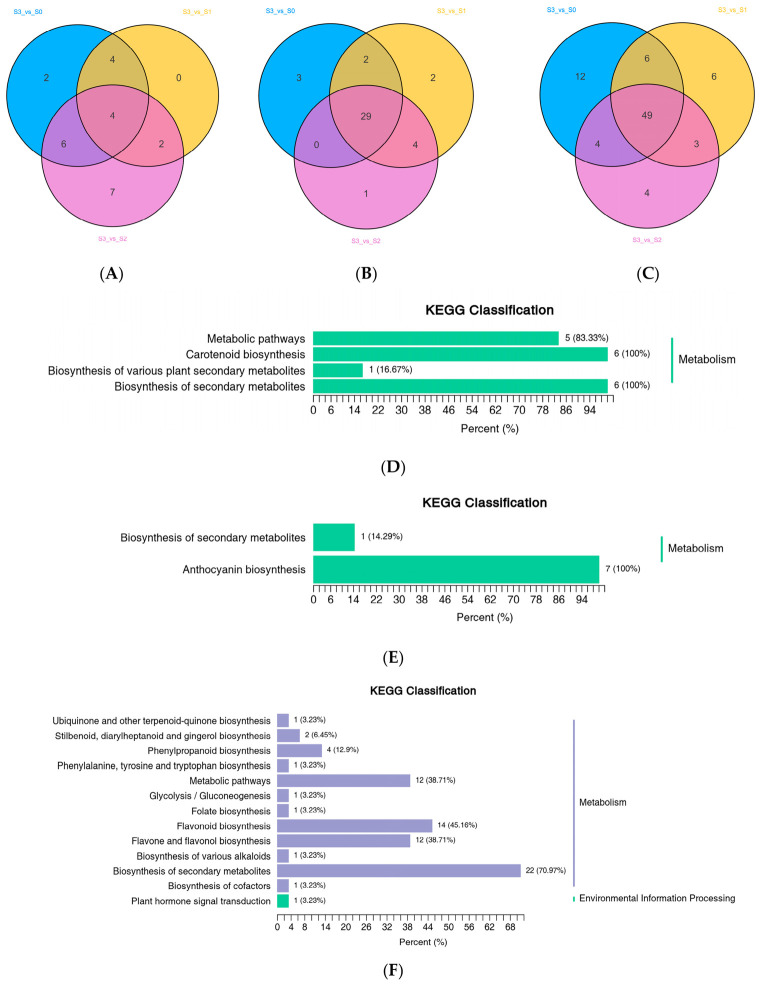
Analysis of differentially accumulated metabolites associated with pigment biosynthesis between the S3 stage and other stages. (**A**) Venn diagram depicting carotenoid-related differentially accumulated metabolites. (**B**) Venn diagram illustrating anthocyanin-related differentially accumulated metabolites. (**C**) Venn diagram showing flavonoid-related differentially accumulated metabolites. (**D**) Carotenoid-related differentially accumulated metabolites KEGG differential enrichment classification analysis. (**E**) Anthocyanin-related differentially accumulated metabolites KEGG differential enrichment classification analysis. (**F**) Flavonoid-related differentially accumulated metabolites KEGG differential enrichment classification analysis. The number in the overlapping area between circles indicates the quantity of differentially expressed metabolites shared among the groups, and the number in the non-overlapping regions represents the quantity of differentially expressed metabolites unique to each respective group.

Employing VIP > 1, |Log2FC| ≥ 1, and *p*-value < 0.05 as the selection criteria, we identified 16 carotenoid metabolites, 36 anthocyanin metabolites, and 69 flavonoid metabolites across the 4 comparisons (Tables S2–S4). Considering that the flower color transition was completed at stage S3 and that this stage showed higher levels and greater diversity of pigment biosynthesis-related accumulated metabolites, we concentrated on comparing the data between S3 and the other stages. The results indicated that, in comparison with other stages, S3 had 25 differentially accumulated carotenoid-related metabolites, 41 differentially accumulated anthocyanin-related metabolites, and 84 differentially accumulated flavonoid metabolites in petals. Among these, the comparison between S3 and S0 exhibited the most significant metabolic differences. We identified 16 differentially expressed carotenoid metabolites (12 down-regulated, 4 up-regulated), 34 differentially expressed anthocyanin metabolites (1 down-regulated, 33 up-regulated), and 71 differentially expressed flavonoid metabolites (31 down-regulated, 40 up-regulated) (Figure 4A–C, Table S5).

Notably, anthocyanin metabolites showed a significant stage-dependent accumulation pattern. Most anthocyanin glycosides rose sharply in S3 relative to S0, S1, and S2. This marked upregulation points to S3 as a key stage for anthocyanin biosynthesis and accumulation. In contrast, carotenoid metabolites accumulated at higher levels in stages S0–S2, followed by a substantial decline in stage S3. This contrasting accumulation pattern between anthocyanins and carotenoids implies a metabolic shift in petal basal markings from an early yellow/orange coloration to a later red/purple hue. Flavonoid differentially accumulated metabolites presented a more intricate pattern, with both up-regulated and down-regulated metabolites being observed across all comparison groups, which reflects the dynamic remodeling of the phenylpropanoid pathway during petal development.

To clarify the biological pathways underlying the aforementioned metabolic transition, a KEGG enrichment analysis was separately conducted on the 3 categories of differentially accumulated metabolites. The results revealed that carotenoid-associated metabolites (Figure 4D) were almost exclusively mapped to carotenoid biosynthesis (100%) and the overarching biosynthesis of secondary metabolites (100%), with a minor fraction falling into biosynthesis of various plant secondary metabolites (16.67%). This strikingly narrow distribution suggests that carotenoid metabolism operates as a relatively independent and self-contained pathway during petal development. Anthocyanin-related metabolites (Figure 4E) displayed an even higher degree of pathway specificity, with anthocyanin biosynthesis accounting for 100% of the annotated hits and only a marginal contribution from biosynthesis of secondary metabolites (14.29%), consistent with the late-stage surge in anthocyanin accumulation. In stark contrast, flavonoid differentially accumulated metabolites (Figure 4F) were distributed across the broadest metabolic network, significantly enriching not only in flavonoid biosynthesis (45.16%) and flavone and flavonol biosynthesis (38.71%) but also in the upstream phenylpropanoid biosynthesis (12.9%). Notably, these flavonoid-associated metabolites are also connected to peripheral pathways such as glycolysis/gluconeogenesis, folate biosynthesis, and plant hormone signal transduction, implicating flavonoid metabolism as a central hub that integrates carbon metabolism and developmental signaling during petal color transition. Despite these divergent pathway profiles, biosynthesis of secondary metabolites was represented across all 3 metabolite classes, albeit at markedly different proportions (100%, 14.29%, and 70.97%, respectively), suggesting a global but differentially weighted activation of secondary metabolism during ontogenetic color change. Collectively, these enrichment patterns reveal a clear metabolic partitioning: The carotenoid and anthocyanin pathways operate as relatively discrete, stage-specific modules, whereas the flavonoid network occupies a more central and interconnected position, potentially bridging the transition from early carotenoid-dominated pigmentation to late-stage anthocyanin accumulation.

### 3.3. Assembly and Functional Annotation of the Petal Transcriptome

**Figure 5:**
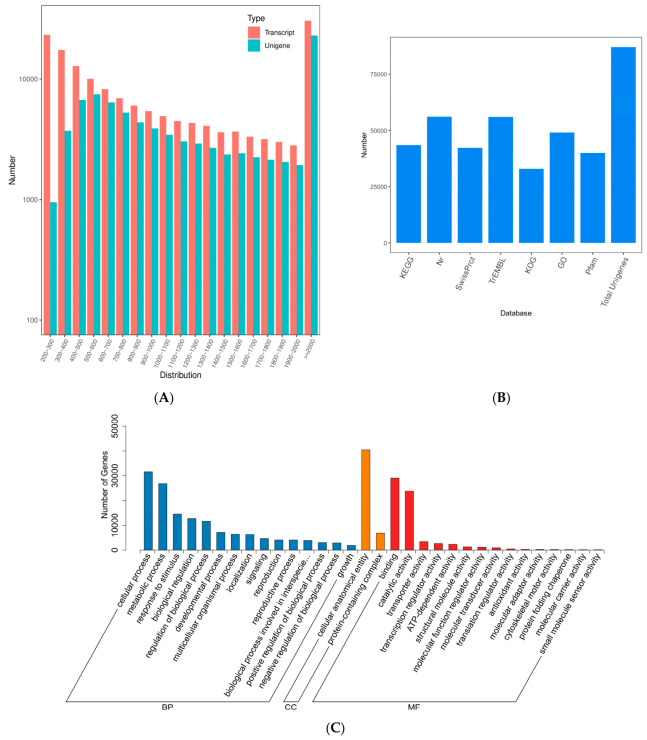
Statistics of transcriptome assembly and annotation for *W. japonica*. (**A**) Distribution of transcript and unigene lengths; (**B**) Annotation statistics across multiple databases; (**C**) GO functional classification.

To elucidate the molecular mechanisms of petal pigment metabolism in *W. japonica* at the transcriptional level, de novo transcriptome sequencing was conducted on 12 samples spanning four developmental stages (S0, S1, S2, and S3). Following quality control and filtering, a total of 93.51 Gb of clean data were acquired, with each sample generating over 6 Gb of clean reads. The Q30 base percentage surpassed 96% for all samples, and the GC content distribution was normal, suggesting that the sequencing data fulfilled the requirements for subsequent analyses. The clean reads were assembled using Trinity software, and after redundancy elimination, a total of 158,199 transcripts and 86,984 unigenes were obtained. Length distribution analysis indicated an average transcript length of 1225 bp and an average unigene length of 1542 bp, with the majority of sequences concentrated between 200 and 2000 bp. Transcripts and unigenes longer than 2000 bp accounted for the highest proportion (Figure 5A).

Unigenes were annotated through comparison with public databases, namely NR, Swiss-Prot, KEGG, KOG, GO, Pfam, and TrEMBL. The results showed that a total of 56,992 Unigenes (65.52%) received annotation in at least one database. The highest annotation rate was found in the NR database (64.35%), followed by TrEMBL (64.26%), GO (56.41%), and KEGG (49.88%) (Table 1, Figure 5B). GO functional classification disclosed that the annotated genes were predominantly enriched in categories such as metabolic process, catalytic activity, cellular process, and binding (Figure 5C). The KOG classification showed that the DEGs were mainly engaged in general functional prediction, signal transduction mechanisms, post-translational modification, and carbohydrate transport and metabolism. Moreover, over 1500 transcription factors were predicted among all the annotated genes, encompassing major families like bHLH, AP2/ERF, WRKY, NAC, and MYB, which laid the groundwork for subsequent regulatory network analysis (Figure S3).

### 3.4. Differential Expression Reflects Ordered Activation of Pigment Biosynthesis Pathways

FPKM values computed based on RSEM were employed for the screening of DEGs using the DESeq2 software, with |log_2_FoldChange| ≥ 1 and FDR < 0.05 serving as the thresholds. The statistical outcomes of differentially expressed genes across each comparison group are presented in Table 2. The S3 vs. S0 comparison group exhibited the highest quantity of DEGs, amounting to 20,073 (8963 up-regulated and 11,110 down-regulated), this was followed by S2 vs. S0 (14,077) and S3 vs. sS1 (14,151). The S2 vs. S1 comparison group had the smallest number of differentially expressed genes, with 5419 (2072 up-regulated and 3347 down-regulated). Volcano plots showed that the log_2_FoldChange values of differentially expressed genes in all comparison groups were predominantly concentrated within the ±5 range, whereas the −log_10_(FDR) values were between 0 and 10 (Figure S4).

GO enrichment of DEGs revealed that, in each comparison group, the most prominent biological processes were cellular processes, metabolic processes, biological regulation, and stress response. Enriched cellular component categories included cellular anatomical entities and intracellular components. Molecular functions such as catalytic activity and binding were also prominent (Figure S5A). KEGG analysis linked DEGs across all groups to multiple pigment metabolism pathways, including flavonoid, anthocyanin, phenylpropanoid, and carotenoid biosynthesis, plus plant hormone signal transduction. The bubble plot highlighted significant anthocyanin biosynthesis enrichment in S3 vs. S0, S3 vs. S1, and S3 vs. S2, underscoring its importance during late-stage petal coloration in *W. japonica*. Plant circadian rhythm and starch and sucrose metabolism were additionally enriched across several groups. The breadth of these enrichments suggests that petal development and pigment accumulation rely on multiple pathways working in concert (Figure S5B).

### 3.5. Integrated Multi-Omics Analysis Decodes the Temporal Relay of Pigment Biosynthesis

**Figure 6:**
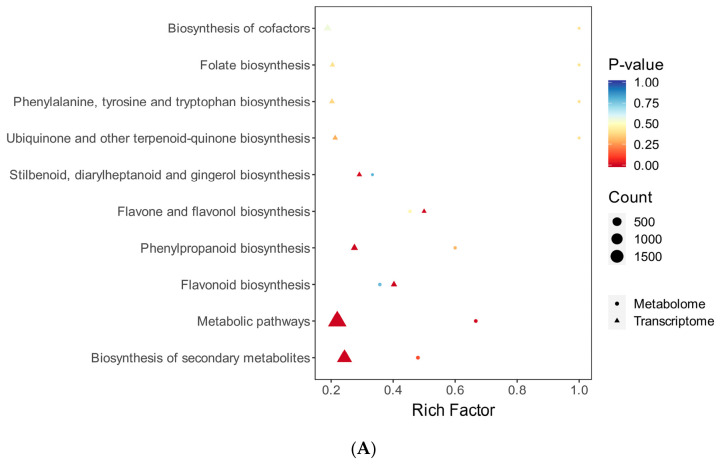

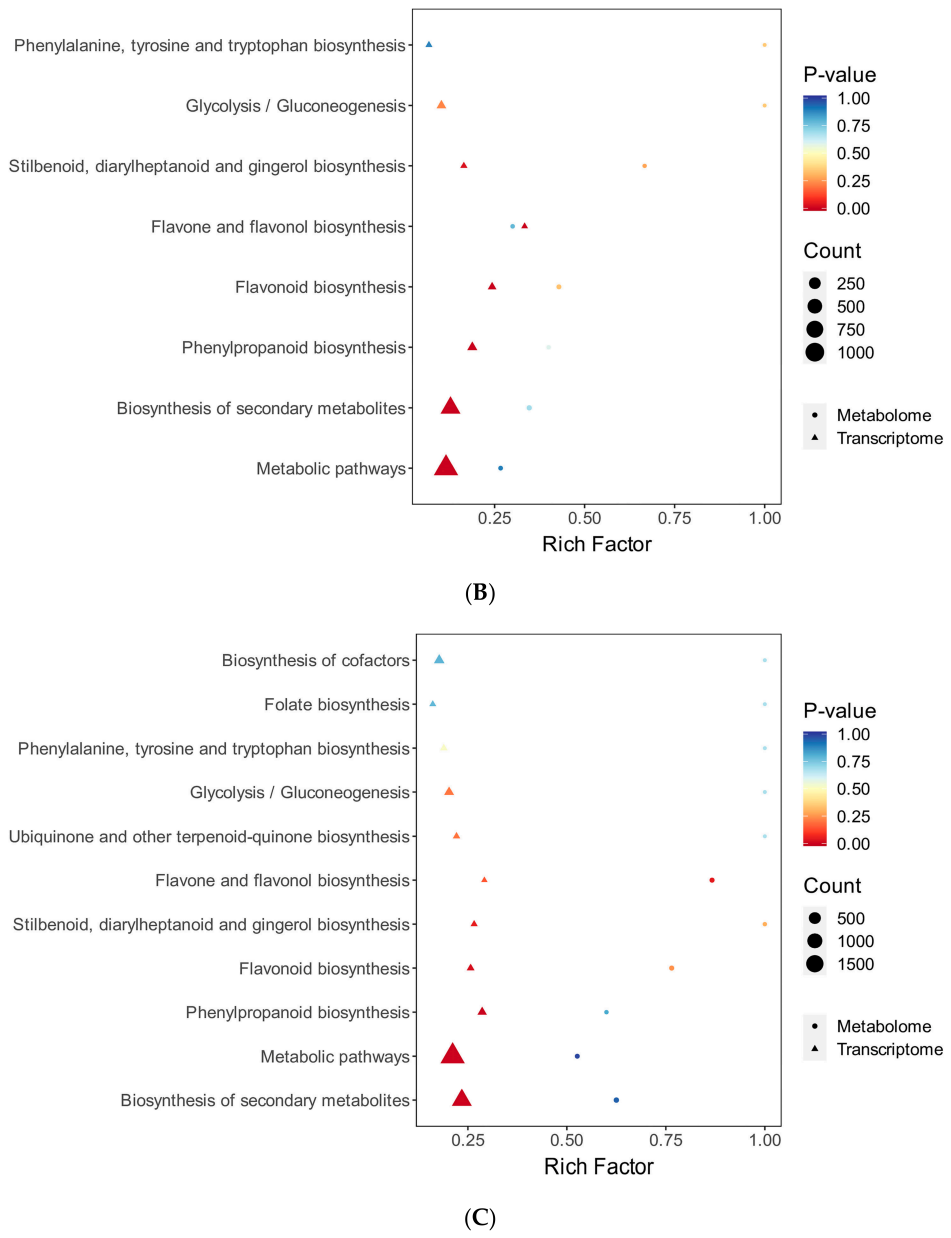
Integrated KEGG pathway enrichment analysis of metabolomics and transcriptomics at different stages. (**A**) Bubble plot of KEGG pathway enrichment analysis for metabolomics and transcriptomics in S1 stage. (**B**) Bubble plot of KEGG pathway enrichment analysis for metabolomics and transcriptomics in S2 stage. (**C**) Bubble plot of KEGG pathway enrichment analysis for metabolomics and transcriptomics in S3 stage.

To deeply elucidate the molecular regulatory mechanisms underlying the color transition of *W. japonica* petal basal markings from initial yellow to yellow-orange and to final purple-red, this study conducted an integrated analysis of metabolomics and transcriptomics data. KEGG pathway enrichment analysis revealed dynamic changes in pigment metabolic pathways at different flower color stages, while qPCR experiments validated the key structural genes and transcription factors involved in petal pigmentation. During the S0 to S1 integrated metabolomics and transcriptomics analysis identified 10 commonly enriched pathways (Figure 6A), primarily including flavonoid biosynthesis, metabolic pathways, secondary metabolite biosynthesis, flavonol biosynthesis, and phenylpropanoid biosynthesis. Among these, the phenylpropanoid biosynthesis pathway, serving as an upstream route for both flavonoid and anthocyanin metabolism, was significantly enriched at this stage, indicating that pigment synthesis in petals had already been initiated during the yellow formation phase. The transcriptome data revealed that in the flavonoid biosynthesis pathway, genes related to *CHS* were significantly up-regulated, laying the foundation for subsequent accumulation of flavonoids. During the transition from S2 to S1, an integrated metabolomics and transcriptomics analysis identified eight commonly enriched pathways (Figure 6B). In comparison with the previous stage, the enrichment levels of the phenylpropanoid biosynthesis and flavonoid biosynthesis pathways increased further, whereas the glycolysis/gluconeogenesis pathway exhibited new enrichment, suggesting active energy metabolism that supplies sufficient carbon sources and energy for pigment synthesis. At this stage, the carotenoid metabolism-related pathways started to be significantly enriched, which is consistent with the phenotypic change in the petal basal markings turning yellow-orange. During the transition stage from S3 to S2, an integrated metabolomics and transcriptomics analysis identified 11 commonly enriched pathways (Figure 6C). At this stage, the biosynthesis pathways of phenylpropanoids, flavonoids, and flavonols exhibited the highest enrichment levels. Meanwhile, the plant hormone signal transduction pathway was also significantly enriched, suggesting a crucial role of hormonal regulation in the final color determination of flowers. The transcriptome data also showed that key enzyme genes involved in the anthocyanin biosynthesis pathway, such *DFR* and anthocyanidin synthase (*ANS*), were significantly up-regulated during this period, directly promoting anthocyanin accumulation and leading to the final purple-red petal color.

**Figure 7:**
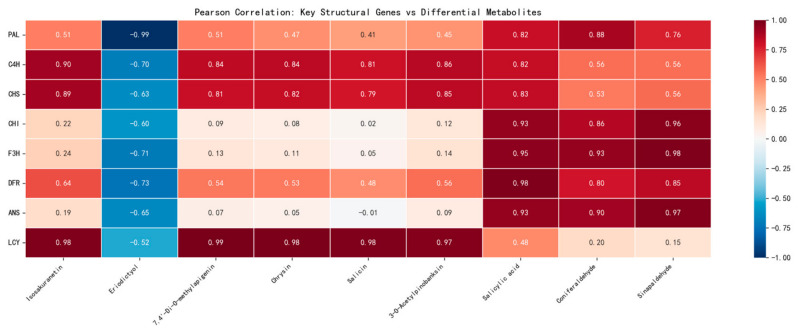
Pearson correlation matrix between the eight key structural genes that showed significant correlations with metabolites (*PAL*, *C4H*, *CHS*, *CHI*, *F3H*, *DFR*, *ANS*, *LCY*) and the ten differentially accumulated metabolites with the strongest correlations. Red indicates positive correlation, blue indicates negative correlation. The dendrogram clustering reveals two modules: an upstream phenylpropanoid cluster (upper-left, PAL/C4H-centered) linked to early flavonoid intermediates, and a downstream anthocyanin cluster (lower-right, DFR/ANS-centered) associated with flavonol glycosides. Flavonoid intermediates (eriodictyol) occupy bridging positions between the two modules, consistent with their metabolic hub function.

To establish direct functional links between the transcriptional regulation and metabolic phenotypes described above, Pearson correlation analysis was performed between the expression levels (FPKM) of eight key structural genes (*PAL*, *C4H*, *CHS*, *CHI*, *F3H*, *DFR*, *ANS*, and *LCY*) and the accumulation levels of 58 differentially accumulated metabolites across all four developmental stages (S0–S3). Among the ten genes selected for subsequent qRT-PCR validation, *PSY* and *ZDS* were excluded from this analysis because they did not exhibit significant correlations (|r| < 0.8) with the metabolite panel, which is dominated by flavonoid and anthocyanin compounds. This absence reflects the pathway separation between carotenoid and flavonoid metabolism during early-stage pigmentation: *PSY* and *ZDS* operate in the carotenoid pathway, whereas the differentially accumulated metabolites are predominantly derived from the phenylpropanoid-flavonoid branch. The correlation matrix (Figure 7) revealed that the eight correlated genes formed two distinct clusters: an upstream phenylpropanoid cluster (*PAL*, *C4H*) linked to early flavonoid intermediates, and a downstream anthocyanin cluster (*DFR*, *ANS*) associated with flavonol glycosides. This clustering pattern supports the sequential activation model, in which transcriptional activation proceeds along the biochemical pathway in a stepwise manner rather than through independent regulatory events. The number of significant correlations per gene is summarized in Table 3.

Among the eight genes, *CHS* showed the highest number of significant correlations (n = 289, Table 3), which is consistent with its position as the entry enzyme of flavonoid biosynthesis. Because *CHS* catalyzes the first committed condensation step, it draws carbon flux from multiple phenylpropanoid precursors and therefore exhibits broad metabolic connectivity. Its strongest correlation with isosakuranetin (r = 0.996, *p* < 1 × 10^−11^) (Table 4) suggests that *CHS* expression is the rate-limiting determinant of flavanone accumulation under our experimental conditions, with minimal interference from competing pathways. The terminal genes *DFR* and *ANS* exhibited strong correlations with eriodictyol (r = 0.990 and 0.993, respectively, Table 4), a dihydroflavonol that serves as their direct substrate. The slightly stronger correlation for ANS than for *DFR* indicates that the oxidation step may become rate-limiting during the final purple-red stage (S3), when anthocyanin accumulation surges. This interpretation aligns with the temporal expression pattern, in which ANS peaks at S3 while *DFR* is already elevated at S2. The strongest correlations for each gene are listed in Table 4.

**Figure 8:**
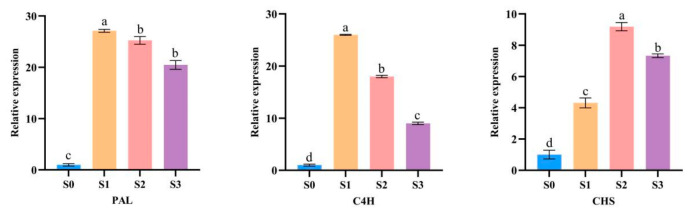

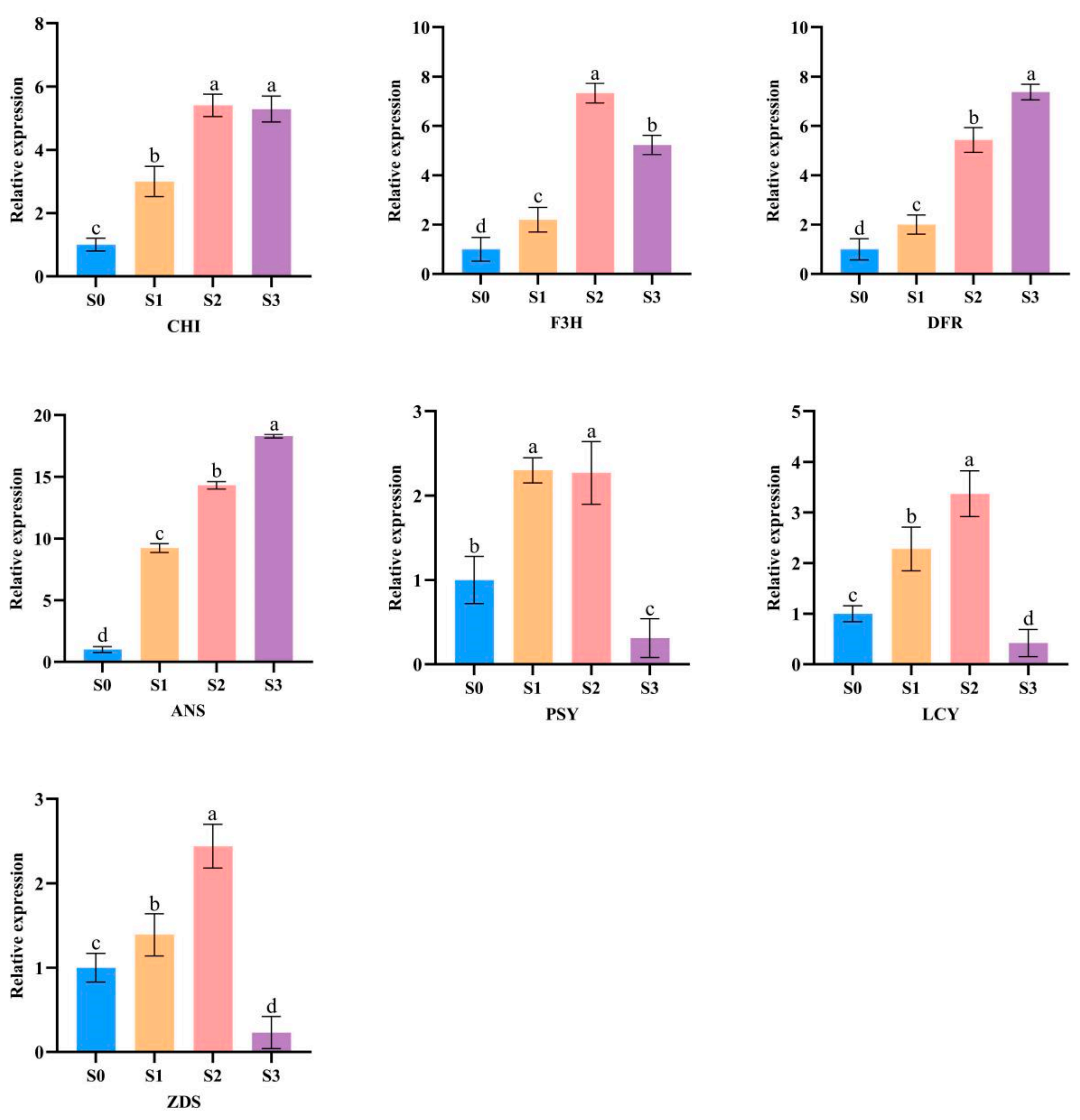
Relative expression of key genes associated with flower color at different flowering stages in *W. japonica*. Three biological replicates were set up. One-way analysis of variance (ANOVA) and Duncan’s method were used to analyze the significance of data differences. The different letters within a column marked significant differences (*p* < 0.05).

The correlation matrix further revealed that the phenylpropanoid-flavonoid cluster and the carotenoid-linked cluster (*LCY*) were largely uncorrelated with each other (Figure 8), reinforcing the interpretation that these two pigment pathways operate as temporally discrete modules. Flavonoid intermediates (notably eriodictyol) occupied bridging positions between the early and late clusters, consistent with their role as metabolic hub intermediates that channel carbon flux from the phenylpropanoid pool toward anthocyanin accumulation during S3.

To validate the transcriptome data, we selected 10 key genes associated with flower color for qPCR verification. These genes include phenylpropanoid pathway genes *PAL* and *C4H*, flavonoid biosynthesis genes *CHS*, *CHI*, and *F3H*, anthocyanin synthesis genes *DFR* and *ANS*, and carotenoid biosynthesis genes *PSY*, *LCY*, and *ZDS*. The qPCR results indicated that *PAL* and *C4H* showed significantly elevated expression during the initial stage. This is consistent with the significant enrichment of the phenylpropanoid pathway observed in the integrated analysis at this stage, suggesting that the synthesis of phenylpropanoid compounds is activated early in the color initiation phase. *CHS*, *CHI*, and *F3H* attained high expression levels during the yellow-to-yellow-orange transition stage, facilitating the accumulation of flavonoids. *DFR* and *ANS* reached their peak expression during the yellow-orange-to-purple-red transition stage, directly promoting anthocyanin synthesis, which was in close agreement with the final purple-red petal phenotype. Carotenoid-related genes *PSY*, *LCY*, and *ZDS* were significantly up-regulated during the yellow-to-yellow-orange stage, consistent with the enrichment of the carotenoid metabolic pathway at this stage, jointly determining the yellow-orange coloration of petals (Figure 8).

Based on the above results, during the color transition of *W. japonica* petal basal markings from the initial yellow to yellow-orange, and ultimately to purple-red, the phenylpropanoid biosynthesis, flavonoid biosynthesis, and carotenoid metabolism pathways are activated in a sequential manner. *PAL* and *C4H* are initially activated during the yellow stage, supplying precursors for subsequent pigment synthesis. *CHS*, *CHI*, and *F3H* are notably up-regulated during the yellow to yellow-orange stage, facilitating the accumulation of flavonoids. *DFR* and *ANS* attain peak expression during the yellow-orange to purple-red stage, directly promoting anthocyanin synthesis. Meanwhile, carotenoid-related genes such as *PSY*, *LCY*, and *ZDS* exhibit coordinated changes, jointly determining the final petal color in conjunction with anthocyanin metabolism.

## 4. Discussion

Flower color change is a prevalent adaptive phenomenon in nature, which has been reported in at least 33 orders, 78 families, and 253 genera of plants. It typically serves to direct pollinators toward newly opened, high-reward flowers. For instance, within the genus *Weigela* (Caprifoliaceae), *W. middendorffiana* alters its flower color from white to red, thereby reducing self-pollination by bumblebees [42]. *W. japonica* experiences a petal basal marking color shift from yellow to purple-red within 4 days after opening. With high ornamental value and strong resistance, it has become a premium landscape tree species in northeastern China [2]. Therefore, this study combines metabolomics and transcriptomics analyses to clarify the metabolic and molecular basis that underlies the petal basal markings color transition in *W. japonica*—from yellow to yellow-orange, and then to deep purple-red—during its 4-day blooming period.

Flower color in plants is predominantly determined by the differential accumulation of endogenous pigments within petals. The sequential accumulation of carotenoids at S2 followed by anthocyanins at S3 in *W. japonica* represents a temporal relay pattern that differs from the pigment dynamics reported in other ornamental species. In *Lonicera japonica*, the pericarp color transition from green to yellow involves a continuous increase in carotenoids and a concomitant decrease in chlorophyll and anthocyanins, suggesting that carotenoid and anthocyanin pathways are coordinately down-regulated rather than sequentially activated [43]. In *Hibiscus mutabilis*, the white-to-red transition is driven predominantly by a single wave of anthocyanin accumulation, with minimal contribution from carotenoid intermediates [44]. By contrast, *W. japonica* maintains both pigment pathways throughout petal development, with the late-stage cyanidin-3-O-glucoside surge (21.94-fold at S3, as quantified in the metabolomic analysis) acting as the primary driver of the red-purple phenotype while carotenoid levels decline. The coexistence of procyanidin B3 and (-)-catechin alongside anthocyanins at S3 suggests that flavonoid copigmentation may stabilize the anthocyanin chromophore, a mechanism consistent with the color enhancement reported in *Rosa hybrida* but not previously documented in *Weigela* species. However, the relay pattern in *W. japonica* is not merely a passive alternation of pigment accumulation; rather, it suggests an active developmental switching mechanism at the metabolic branch point. The sharp decline in carotenoid levels concurrent with the anthocyanin surge at S3 implies that the metabolic flux may be redirected from the carotenoid pathway to the flavonoid branch via shared precursors or competing enzymatic activities, a phenomenon analogous to the metabolic shift observed during tomato fruit ripening where carotenoid accumulation precedes and potentially suppresses flavonoid synthesis through precursor competition [45,46].

The phenylpropanoid biosynthesis pathway functions as the upstream entry point for the synthesis of flavonoids and anthocyanins [47]. The temporal ordering of structural gene expression in *W. japonica PAL* and *C4H* active at S0–S1, *CHS*/*CHI*/*F3H* peaking at S1–S2, and *DFR*/*ANS* maximal at S2–S3 parallels the sequential relay model proposed for *Rosa damascena*, where *CHS*/*CHI* expression precedes *DFR*/*ANS* activation during petal opening [48]. However, unlike the continuous up-regulation of anthocyanin biosynthetic genes reported in *Prunus persica* fruit ripening [30], where transcripts remain elevated throughout the coloration period, *W. japonica* exhibits a sharp, stage-specific induction of *DFR* and *ANS* exclusively at S3. This developmental gating suggests that the transition from flavonoid to anthocyanin production is controlled by a regulatory switch rather than a gradual gradient. Notably, the *F3H* expression peak at S2, which precedes the *DFR*/*ANS* surge by one developmental stage, indicates a temporal partitioning at the flavonoid branch point: dihydrokaempferol is first converted to dihydroquercetin (via *F3H*) before being channeled to anthocyanin synthesis, a sequence that differs from the overlapping *F3H* and *DFR* expression observed in *Gentiana triflora* where both genes co-accumulate throughout development. The coordinated high expression of carotenoid biosynthesis genes (*PSY*, *LCY*, *ZDS*) from S1 to S2, concurrent with the *CHS*/*CHI*/*F3H* peak, suggests that the two pigment pathways are transcriptionally coordinated but not competitively inhibited during the yellow-orange stage. The subsequent decline of carotenoid gene expression at S3, coinciding with the *DFR/ANS* surge, implies a possible metabolic competition for shared precursors or a developmental program-driven reallocation of transcriptional resources from the carotenoid to the flavonoid branch. In both the flavonoid and anthocyanin biosynthetic pathways, the temporal expression profiles of structural genes were closely aligned with metabolite accumulation. *CHS*, *CHI*, and *F3H* exhibited significant up-regulation from S1 to S2, while *DFR* and *ANS* reached their maximum expression levels from S2 to S3, directly facilitating large-scale anthocyanin synthesis.

Transcription factors play a pivotal role in the accurate regulation of flower color variation. The MYB-bHLH-WDR ternary complex is recognized as a conserved module for regulating anthocyanin biosynthesis. In this complex, R2R3-MYB determines the regulatory specificity, bHLH offers an interaction platform, and WD40 enhances the stability of the complex. In *Gentiana*, the spatiotemporal expression of GtMYB3 and GtbHLH1 is closely associated with anthocyanin accumulation and the expression of structural genes [49]. In *Centaurea cyanus*, CcMYB6-1 and CcbHLH1 synergistically initiate anthocyanin synthesis [50]. The WGCNA identified MYB44, bHLH92, and ERF3 as hub genes strongly correlated with the S3 anthocyanin surge (turquoise module correlation coefficient r = 0.94), suggesting that these transcription factors may function as developmental switches for the late-stage color transition. In *Centaurea cyanus*, CcMYB6-1 activation precedes anthocyanin accumulation by approximately two developmental stages, suggesting an early priming role for MYB factors. In *W. japonica*, however, the MYB44 expression peak at S2 coincides with the F3H maximum and precedes the DFR/ANS surge by only one stage, implying a tighter temporal coupling between MBW complex formation and anthocyanin synthesis. The ERF3 hub gene, which belongs to the ethylene response factor family, has been associated with ethylene-mediated pigment regulation in *Petunia hybrida* [40]; its strong correlation with the S3 module suggests that ethylene signaling may participate in triggering the late-stage anthocyanin burst, though this hypothesis remains to be tested. It is important to note that the current WGCNA-based analysis identifies statistical associations rather than causal regulatory relationships, and the de novo transcriptome assembly (86,984 unigenes, N50 = 1287 bp) may under-represent low-abundance regulatory transcripts. The significant accumulation of flavonoid metabolites at stage S3 could potentially contribute to anthocyanin stabilization via copigmentation effects, as hypothesized for *Rosa hybrida* [51], but direct evidence for copigmentation complex formation in *W. japonica* is currently lacking. Future validation through gene overexpression, yeast two-hybrid assays, and chromatin immunoprecipitation will be necessary to establish the causal regulatory relationships implied by the current correlation-based analysis.

## 5. Conclusions

This study suggests that the rapid ontogenetic color transition of *W. japonica* petal basal markings follows a sequential activation model in which carotenoids, flavonoids, and anthocyanins accumulate in a temporally partitioned manner. The integration of metabolomic and transcriptomic data is consistent with coordinated shifts in pigment metabolism and structural gene expression across developmental stages, though the causal regulatory relationships remain to be functionally validated.

## Figures and Tables

**Table 1:** Unigene functional annotation statistics.

Database	Number of Annotated Genes	Annotation Rate (%)
NR	55,973	64.35
TrEMBL	55,894	64.26
GO	49,069	56.41
KEGG	43,385	49.88
Swiss-Prot	42,226	48.54
Pfam	39,970	45.95
KOG	32,897	37.82

**Table 2:** Statistics of differentially expressed genes at various stages.

Comparison Group	Total DEGs	Up-Regulated Genes	Down-Regulated Genes
S1 vs. S0	11,831	6148	5683
S2 vs. S0	14,077	6734	7343
S2 vs. S1	5419	2072	3347
S3 vs. S0	20,073	8963	11,110
S3 vs. S1	14,151	6164	7987
S3 vs. S2	11,743	4989	6754

**Table 3:** Significant Gene-Metabolite Correlations.

Gene	Pathway	Quantity of Important Metabolites	Mean r	Max r
*PAL*	Phenylpropanoid	173	−0.182	0.994
*C4H*	Phenylpropanoid	75	0.309	0.945
*CHS*	Flavonoid/anthocyanin	289	0.272	0.996
*CHI*	Flavonoid/anthocyanin	8	0.931	0.981
*F3H*	Flavonoid/anthocyanin	8	0.937	0.980
*DFR*	Anthocyanin	77	0.171	0.990
*ANS*	Anthocyanin	26	0.508	0.993
*LCY*	Carotenoid	42	0.367	0.992

**Table 4:** Representative Significant Gene-Metabolite Correlations.

Gene	Metabolite	r	*p*	Class
*CHS*	Isosakuranetin	0.996	<1 × 10^−11^	Flavanone
*PAL*	Eriodictyol	0.994	<1 × 10^−10^	Flavonol
*ANS*	Eriodictyol	0.993	<1 × 10^−10^	Flavonol
*LCY*	Flavonoid_11	0.992	<1 × 10^−10^	Flavonoid
*CHS*	Chrysin	0.992	<1 × 10^−10^	Flavone
*DFR*	Eriodictyol	0.990	<1 × 10^−10^	Flavonol
*C4H*	Chrysoeriol	0.945	<1 × 10^−5^	Flavone
*C4H*	Diosmetin	0.945	<1 × 10^−5^	Flavone
*F3H*	Sinapaldehyde	0.980	<1 × 10^−8^	Phenylpropanoid
*CHI*	Flavonoid_162	0.981	<1 × 10^−8^	Flavonoid

## Data Availability

The datasets generated and/or analysed during the current study are available in the NCBI [National Center for Biotechnology Information: https://www.ncbi.nlm.nih.gov, accessed on 14 June 2026] repository, PRJNA1478148.

## Supplementary Materials

The following supporting information can be downloaded at https://www.mdpi.com/article/10.3390/metabo16070511/s1: Figure S1: TIC chromatogram of QC samples for carotenoids, anthocyanins, and flavonoids, Figure S2: Empirical cumulative distribution function analysis, Figure S3: Transcription factor annotation classification statistics pie chart, Figure S4: Volcano plot of differentially expressed genes across each comparison group, Figure S5: GO and KEGG enrichment analysis of DEGs, Table S1: Primers used in the qRT-PCR, Table S2: ANOVA Summary for total metabolite accumulation, Table S3: Carotenoid-related differential accumulation metabolites, Table S4: Anthocyanin-related differentially accumulated metabolites, Table S5: Flavonoid differential accumulation metabolites, Table S6: Metabolic differences accumulated between the S3 stage and other stages.

## Abbreviations

The following abbreviations are used in this manuscript:

4CL	4-coumarate:CoA ligase
*ANS*	anthocyanin synthase
APCI	atmospheric pressure chemical ionization
BHT	butylated hydroxytoluene
*C4H*	cinnamate 4-hydroxylase
CDS	coding sequences
*CHI*	chalcone isomerase
*CHS*	chalcone synthase
CV	coefficient of variation
DEGs	differentially expressed genes
*DFR*	dihydroflavonol 4-reductase
ECDF	empirical cumulative distribution function
ESI	electrospray ionization
*F3′H*	flavonoid 3′-hydroxylase
*F3H*	flavanone 3-hydroxylase
FDR	false discovery rate
FPKM	fragments per kilobase of transcript per million mapped reads
HCA	hierarchical cluster analysis
*LCY*	lycopene cyclase
MBW	MYB-bHLH-WDR
MRM	multiple reaction monitoring
OPLS-DA	orthogonal partial least squares discriminant analysis
*PAL*	phenylalanine ammonia-lyase
*PSY*	phytoene synthase
QC	quality control
TIC	total ion chromatogram
UPLC-MS/MS	ultra-performance liquid chromatography coupled with tandem mass spectrometry
*ZDS*	zeta-carotene desaturase
Z-score	standard score
