# Supplementary material for: Temporal Partitioning of Carotenoid, Flavonoid and Anthocyanin Biosynthesis Underlies the Ontogenetic Petal Color Transition in Weigela japonica
Source: Metabolites. 2026 Jul 22;16(7):511. doi: 10.3390/metabo16070511 (PMC13413832; doi:10.3390/metabo16070511)
Supplement: Supplementary file 1 [file metabolites-16-00511-s001.zip › Supplementary Materials DOC version (including figure legend).pdf]

Supplementary Materials

A

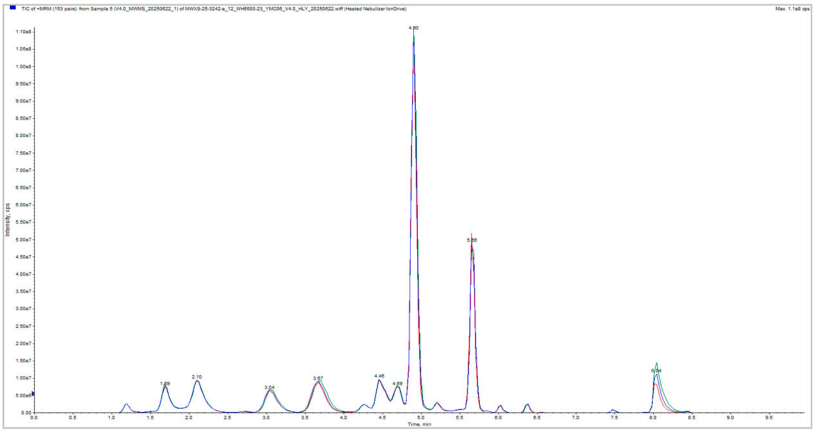

B

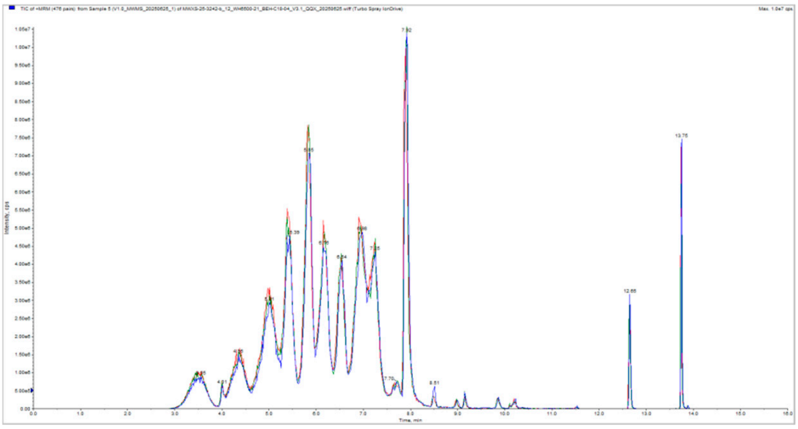

C

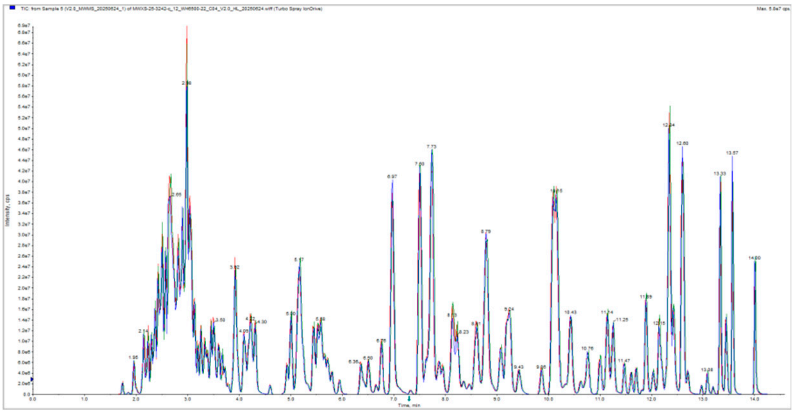

Figure S1 TIC chromatogram of QC samples for carotenoids, anthocyanins, and flavonoids. **(A)** TIC chromatogram of carotenoid QC sample. **(B)** TIC chromatogram of anthocyanin QC sample. **(C)** TIC chromatogram of QC sample for flavonoids. The high overlap of total ion current curves in metabolite detection, which indicates consistent retention times and peak intensities, suggests good signal stability of the mass spectrometer when analyzing the same sample at different times.

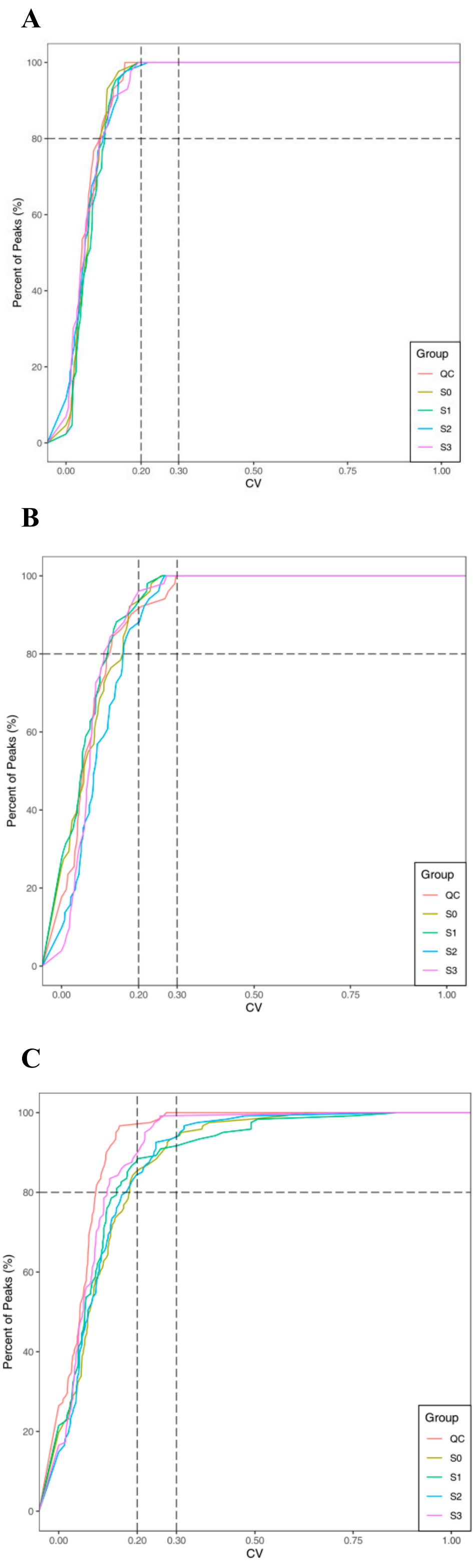

Figure S2 Empirical cumulative distribution function analysis. **(A)** Empirical cumulative distribution function analysis of carotenoids. **(B)** Empirical cumulative distribution function analysis of anthocyanins. **(C)** Empirical cumulative distribution function analysis of flavonoids. The higher the proportion of substances with low coefficient of variation (CV) values in quality control (QC) samples, the more stable the experimental data. When more than 80% of the substances in QC samples have a CV value below 0.3, the data are regarded as stable; when more

than 80% have a CV value below 0.2, the data are regarded as highly stable.

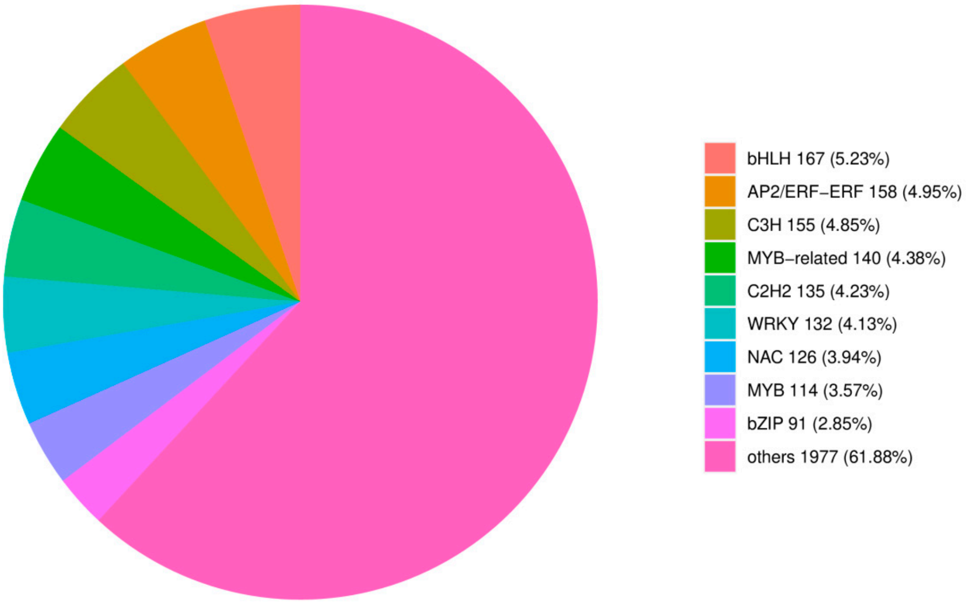

Figure S3 Transcription factor annotation classification statistics pie chart. Different colored squares represent transcription factors from different families.

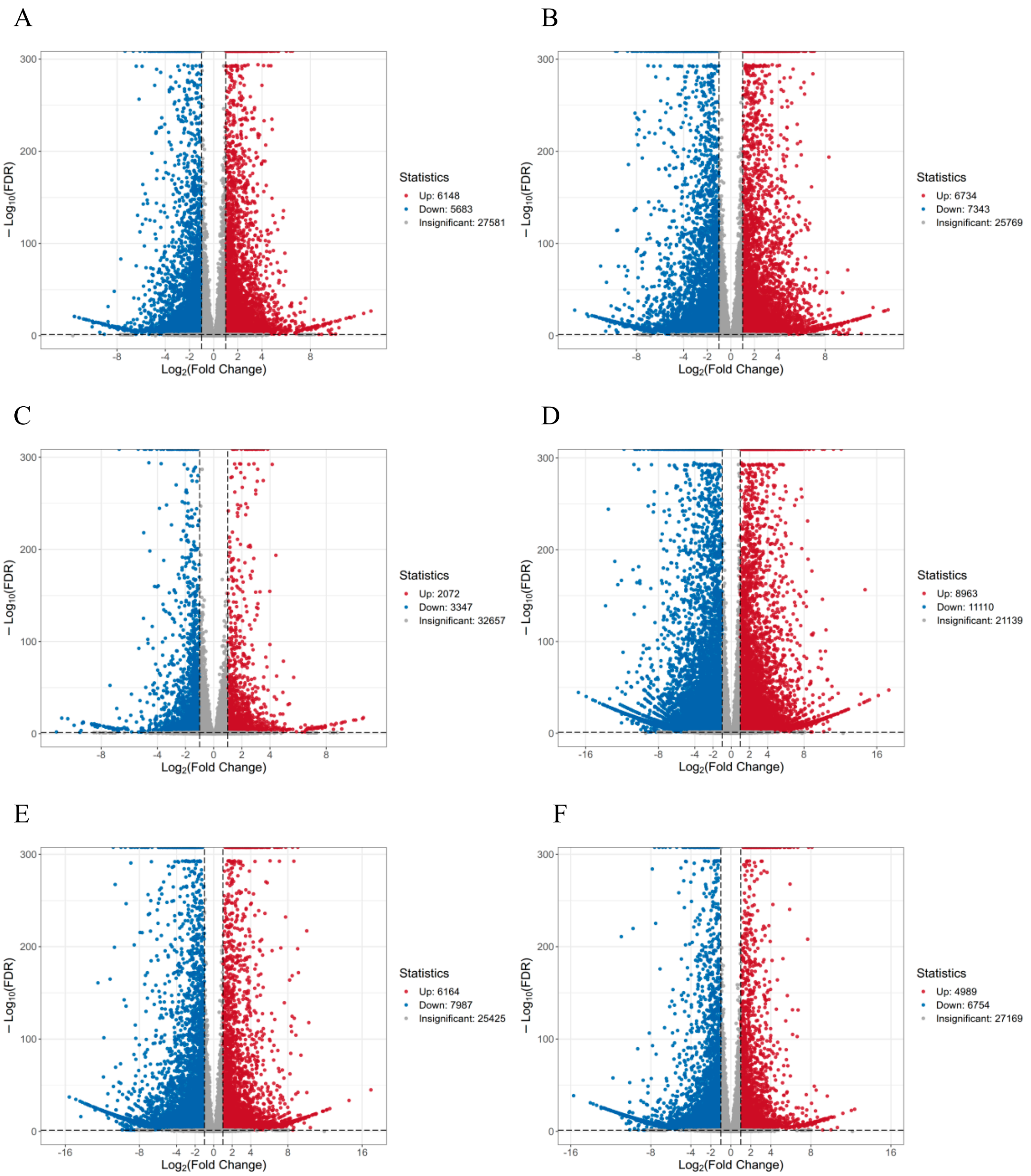

Figure S4 Volcano plot of differentially expressed genes across each comparison group. **(A)**S1vsS0 **(B)**S2vsS0 **(C)** S2vsS1 **(D)** S3vsS0 **(E)** S3vsS1 **(F)**S3vsS2

A

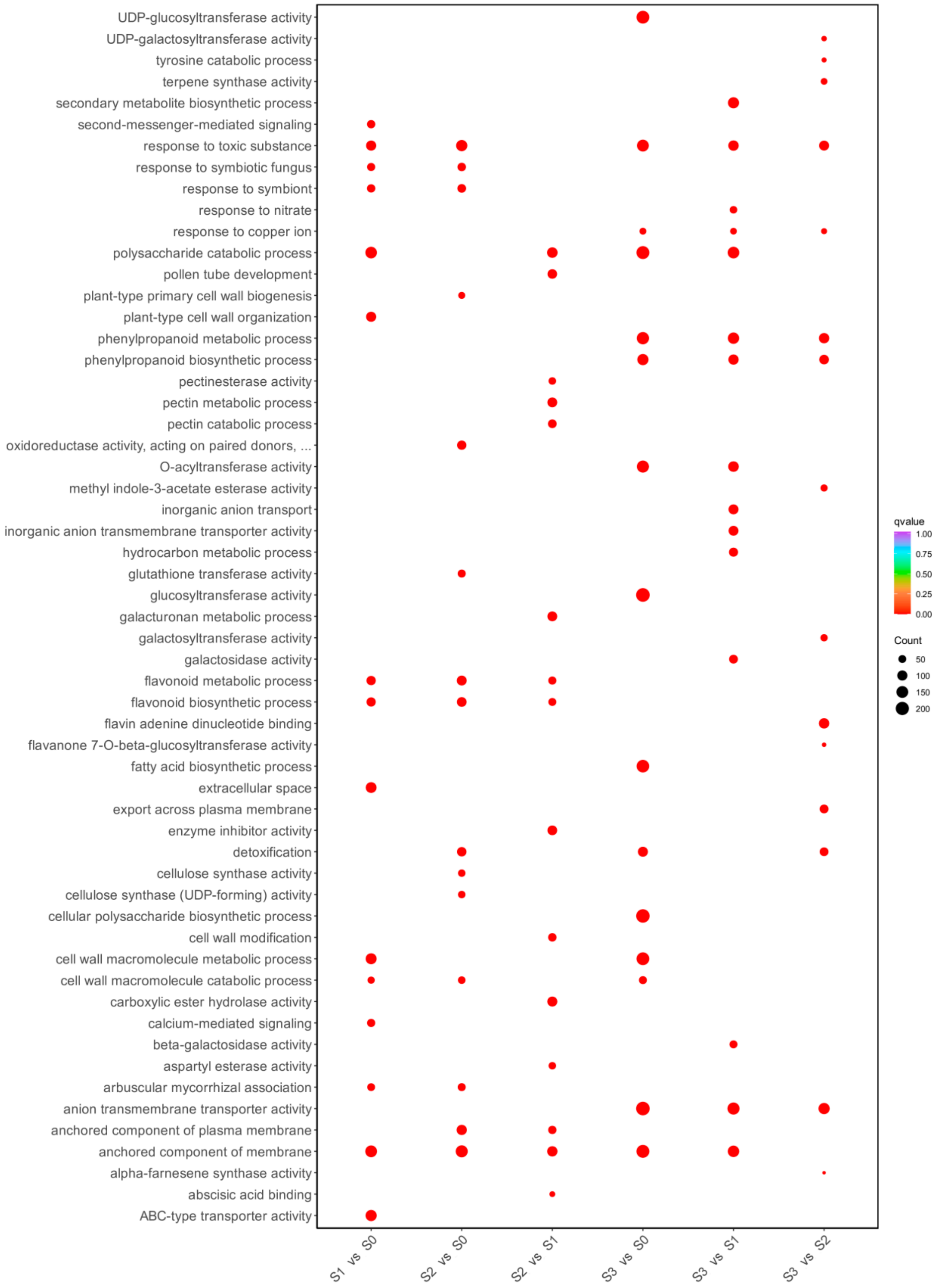
UDP-glucosyltransferase activity
UDP-galactosyltransferase activity
tyrosine catabolic process
terpene synthase activity
secondary metabolite biosynthetic process
second-messenger-mediated signaling
response to toxic substance
response to symbiotic fungus
response to symbiont
response to nitrate
response to copper ion
polysaccharide catabolic process
pollen tube development
plant-type primary cell wall biogenesis
plant-type cell wall organization
phenylpropanoid metabolic process
phenylpropanoid biosynthetic process
pectinesterase activity
pectin metabolic process
pectin catabolic process
oxidoreductase activity, acting on paired donors, ...
O-acyltransferase activity
methyl indole-3-acetate esterase activity
inorganic anion transport
inorganic anion transmembrane transporter activity
hydrocarbon metabolic process
glutathione transferase activity
glucosyltransferase activity
galacturonan metabolic process
galactosyltransferase activity
galactosidase activity
flavonoid metabolic process
flavonoid biosynthetic process
flavin adenine dinucleotide binding
flavanone 7-O-beta-glucosyltransferase activity
fatty acid biosynthetic process
extracellular space
export across plasma membrane
enzyme inhibitor activity
detoxification
cellulose synthase activity
cellulose synthase (UDP-forming) activity
cellular polysaccharide biosynthetic process
cell wall modification
cell wall macromolecule metabolic process
cell wall macromolecule catabolic process
carboxylic ester hydrolase activity
calcium-mediated signaling
beta-galactosidase activity
aspartyl esterase activity
arbuscular mycorrhizal association
anion transmembrane transporter activity
anchored component of plasma membrane
anchored component of membrane
alpha-farnesene synthase activity
abscisic acid binding
ABC-type transporter activity
S1 vs S0
S2 vs S0
S2 vs S1
S3 vs S0
S3 vs S1
S3 vs S2
qvalue
1.00
0.75
0.50
0.25
0.00
Count
50
100
150
200

B

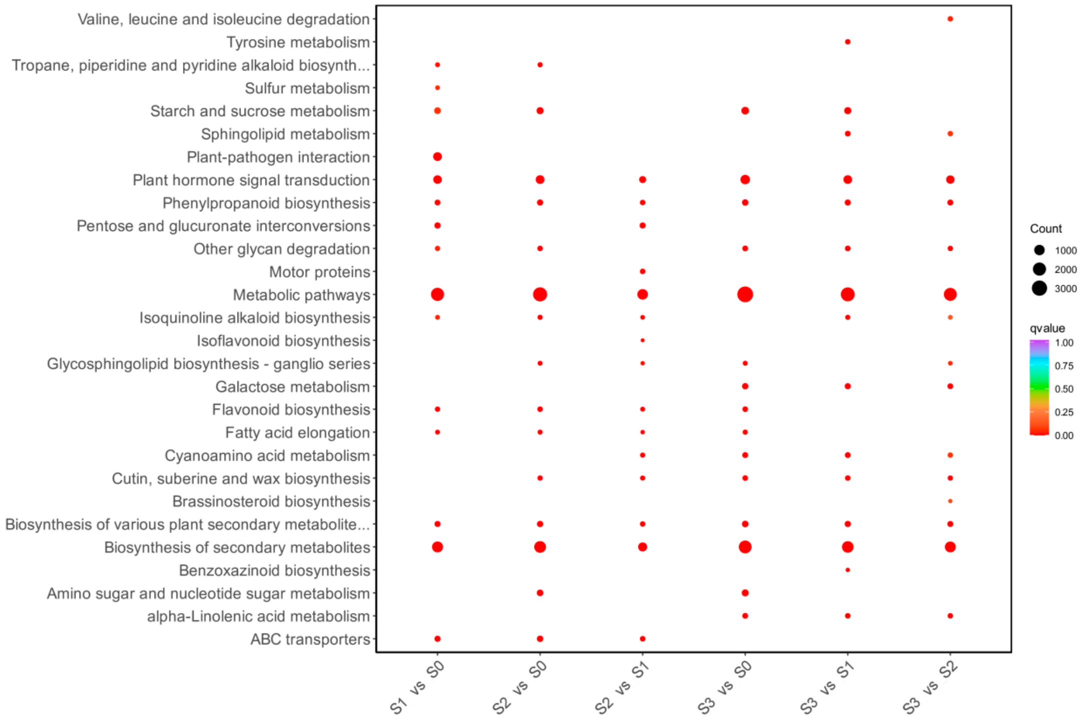

Figure S5 GO and KEGG enrichment analysis of DEGs. **(A)**GO enrichment analysis of DEGs. **(B)**KEGG enrichment analysis of DEGs.

Table S1 Carotenoid-related differential accumulation metabolites

ID	Name	Class
Carotenoid_04	β-carotene	carotenes
Carotenoid_06	(E/Z)-phytoene	carotenes
Carotenoid_15	lutein dilaurate	xanthophylls
Carotenoid_19	lutein distearate	xanthophylls
Carotenoid_28	violaxanthin laurate	xanthophylls
Carotenoid_34	violaxanthin-myristate-laurate	xanthophylls
Carotenoid_45	zeaxanthin dimyristate	xanthophylls
Carotenoid_46	zeaxanthin-laurate-palmitate	xanthophylls
Carotenoid_48	zeaxanthin dipalmitate	xanthophylls
Carotenoid_52	β-cryptoxanthin myristate	xanthophylls
Carotenoid_54	β-cryptoxanthin oleate	xanthophylls
Carotenoid_55	antheraxanthin	xanthophylls
Carotenoid_56	zeaxanthin	xanthophylls
Carotenoid_58	neoxanthin	xanthophylls
Carotenoid_59	lutein	xanthophylls
Carotenoid_61	astaxanthin	xanthophylls

Table S2 Anthocyanin-related differentially accumulated metabolites

ID	Name	Class
Anthocyanidin_04	Cyanidin-3,5-O-diglucoside	Cyanidin
Anthocyanidin_05	Cyanidin-3-O-(6-O-malonyl-beta-D-glucoside)	Cyanidin
Anthocyanidin_07	Cyanidin-3-O-5-O-(6-O-coumaroyl)-diglucoside	Cyanidin
Anthocyanidin_08	Cyanidin-3-O-arabinoside	Cyanidin
Anthocyanidin_10	Cyanidin-3-O-galactoside	Cyanidin
Anthocyanidin_11	Cyanidin-3-O-glucoside	Cyanidin
Anthocyanidin_12	Cyanidin-3-O-rutinoside	Cyanidin
Anthocyanidin_29	Delphinidin-3-O-rutinoside	Delphinidin
Anthocyanidin_32	Delphinidin-3-O-rutinoside-5-O-glucoside	Delphinidin
Anthocyanidin_50	Malvidin-3-O-galactoside	Malvidin
Anthocyanidin_51	Malvidin-3-O-glucoside	Malvidin
Anthocyanidin_66	Pelargonidin-3-O-arabinoside	Pelargonidin
Anthocyanidin_67	Pelargonidin-3-O-galactoside	Pelargonidin
Anthocyanidin_78	Peonidin-3,5-O-diglucoside	Peonidin
Anthocyanidin_85	Peonidin-3-O-galactoside	Peonidin
Anthocyanidin_86	Peonidin-3-O-glucoside	Peonidin
Anthocyanidin_88	Peonidin-3-O-rutinoside	Peonidin
Anthocyanidin_104	Procyanidin A2	Procyanidin
Anthocyanidin_105	Procyanidin B1	Procyanidin
Anthocyanidin_106	Procyanidin B2	Procyanidin
Anthocyanidin_107	Procyanidin B3	Procyanidin
Anthocyanidin_108	Procyanidin C1	Procyanidin
Anthocyanidin_116	Cyanidin-3-xylosyl-galactoside	Cyanidin
Anthocyanidin_117	Cyanidin-3-O-arabinosidase-glucoside	Cyanidin
Anthocyanidin_123	Cyanidin-3-gentiobioside	Cyanidin
Anthocyanidin_134	Cyanidin-3-malonyl-glucosyl-glucoside	Cyanidin
Anthocyanidin_145	Cyanidin-caffeoyl-glucoside-glucoside	Cyanidin
Anthocyanidin_208	Delphinidin-3-O-xyloside	Delphinidin
Anthocyanidin_213	Delphinidin-3-O-(6''-O-tartaryl)glucoside	Delphinidin
Anthocyanidin_214	Delphinidin-3-(6''-O-coumaroyl)galactoside	Delphinidin
Anthocyanidin_232	Delphinidin-acetyl-rhamnoside-rutinoside	Delphinidin
Anthocyanidin_236	Delphinidin-caffeoyl-malonyl-malonyl-rhamnoside-rhamnoside	Delphinidin
Anthocyanidin_245	Malvidin-3-O-(6''-O-acetyl)glucoside	Malvidin
Anthocyanidin_278	Pelargonidin-3-O-rhamnoside-5-O-glu	Pelargonidin

ID	Name	Class
	coside	
Anthocyanidin_319	Peonidin-3-O-xyloside	Peonidin
Anthocyanidin_401	Cyanidin-3,5-O-digalactoside	Cyanidin

Table S3 Flavonoid differential accumulation metabolites

ID	Name	Class
Flavonoid_06	Diosmetin	Flavones
Flavonoid_09	Genistein	Isoflavanones
Flavonoid_16	7,4'-Di-O-methylapigenin	Flavones
Flavonoid_23	Quercetin	Flavonols
Flavonoid_40	Dihydromyricetin	Flavanonols
Flavonoid_50	Luteolin	Flavones
Flavonoid_54	Isorhamnetin-3-O-neohespeidoside	Flavonols
Flavonoid_55	Catechin	Flavanols
Flavonoid_56	Taxifolin	Flavanonols
Flavonoid_57	Rutin	Flavonols
Flavonoid_61	Chrysin	Flavones
Flavonoid_65	Phlorizin	Chalcones
Flavonoid_72	Apigenin	Flavones
Flavonoid_74	Schaftoside	Flavone glycosides
Flavonoid_76	Sakuranetin	Flavanones
Flavonoid_79	Ligustroflavone	Phenonic acids
Flavonoid_86	Avicularin	Flavonols
Flavonoid_96	Calycosin-7-O-β-D-glucoside	Isoflavanones
Flavonoid_99	Acacetin	Flavones
Flavonoid_108	Pinocembrin	Flavanones
Flavonoid_118	Narcissin	Flavonols
Flavonoid_126	Eriodictyol	Flavanones
Flavonoid_138	Baimaside	Flavonols
Flavonoid_139	Dihydrokaempferol	Flavanonols
Flavonoid_146	Icariin	Flavonols
Flavonoid_152	Ononin	Isoflavanones
Flavonoid_154	Vitexin	Flavone glycosides
Flavonoid_158	Isosakuranetin	Flavanones
Flavonoid_160	Apigenin 7-glucoside	Flavones
Flavonoid_165	Homoplantaginin	Flavones
Flavonoid_166	Genkwanin	Flavones
Flavonoid_172	Kaempferitrin	Flavonols
Flavonoid_176	Naringenin-7-glucoside	Flavanones
Flavonoid_177	Phloretin	Chalcones
Flavonoid_179	Poncirin	Flavanones
Flavonoid_181	Demethyltexasin	Isoflavanones
Flavonoid_182	2'-Hydroxydaidzein	Isoflavanones
Flavonoid_197	Isorhamnetin 3-O-glucoside	Flavonols
Flavonoid_201	Trilobatin	Chalcones
Flavonoid_204	Linarin	Flavones
Flavonoid_206	Salicylic acid	Benzoic acid and its derivatives
Flavonoid_217	Sophoraflavonoloside	Flavonols

ID	Name	Class
Flavonoid_221	Eupafolin	Flavones
Flavonoid_233	Norartocarpetin	Flavones
Flavonoid_238	Diosmetol 7-glucoside	Flavones
Flavonoid_244	Chlorogenic acid	Phenylpropionic acids
Flavonoid_245	Quercetin 3-O-neohesperidoside	Flavonols
Flavonoid_246	Rutin Trihydrate	Flavonols
Flavonoid_249	Protocatechuic acid	Benzoic acid and its derivatives
Flavonoid_254	Thermopsoside	Flavones
Flavonoid_260	Leucoside	Flavonols
Flavonoid_263	Cryptochlorogenic acid	Phenylpropionic acids
Flavonoid_265	Vicenin 2	Flavones
Flavonoid_267	Morin	Flavonols
Flavonoid_272	Chrysoeriol	Flavones
Flavonoid_276	3-O-Acetylpinobanksin	Flavanonols
Flavonoid_279	Glycitein	Isoflavanones
Flavonoid_281	Hesperetin 7-O-glucoside	Flavanones
Flavonoid_283	Salicin	Glycosides
Flavonoid_300	Piceatannol	Stilbenes
Flavonoid_301	trans-Piceid	Stilbenes
Flavonoid_309	4',7-Di-O-methylnaringenin	Flavanones
Flavonoid_312	Isorhamnetin 3,7-O-diglucoside	Flavonols
Flavonoid_314	Cacticin	Flavonols
Flavonoid_319	Fraxin	Coumarins
Flavonoid_323	Tamarixin	Flavonols
Flavonoid_324	Isorhoifolin	Flavones
Flavonoid_336	Scopoletin	Coumarins
Flavonoid_343	Didymin	Flavanones

Table S4 Metabolic differences accumulated between the S3 stage and other stages

ID	Name	Class	Regulated
Carotenoid_06	(E/Z)-phytoene	carotenes	up
Carotenoid_12	lutein palmitate	xanthophylls	down
Carotenoid_15	lutein dilaurate	xanthophylls	up
Carotenoid_18	lutein dipalmitate	xanthophylls	down
Carotenoid_19	lutein distearate	xanthophylls	down
Carotenoid_30	violaxanthin palmitate	xanthophylls	down
Carotenoid_39	violaxanthin dioleate	xanthophylls	down
Carotenoid_45	zeaxanthin dimyristate	xanthophylls	up
Carotenoid_46	zeaxanthin-laurate-palmitate	xanthophylls	up
Carotenoid_48	zeaxanthin dipalmitate	xanthophylls	down
Carotenoid_54	β-cryptoxanthin oleate	xanthophylls	down
Carotenoid_55	antheraxanthin	xanthophylls	down
Carotenoid_56	zeaxanthin	xanthophylls	down
Carotenoid_58	neoxanthin	xanthophylls	down
Carotenoid_59	lutein	xanthophylls	down
Carotenoid_61	astaxanthin	xanthophylls	down
Anthocyanidin_04	Cyanidin-3,5-O-diglucoside	Cyanidin	up
Anthocyanidin_05	Cyanidin-3-O-(6-O-malonyl-beta-D-glucoside)	Cyanidin	up
Anthocyanidin_07	Cyanidin-3-O-5-O-(6-O-coumaroyl)-diglucoside	Cyanidin	up
Anthocyanidin_08	Cyanidin-3-O-arabinoside	Cyanidin	up
Anthocyanidin_10	Cyanidin-3-O-galactoside	Cyanidin	up
Anthocyanidin_11	Cyanidin-3-O-glucoside	Cyanidin	up
Anthocyanidin_12	Cyanidin-3-O-rutinoside	Cyanidin	up
Anthocyanidin_32	Delphinidin-3-O-rutinoside-5-O-gl ucoside	Delphinidin	up
Anthocyanidin_50	Malvidin-3-O-galactoside	Malvidin	up
Anthocyanidin_51	Malvidin-3-O-glucoside	Malvidin	up
Anthocyanidin_66	Pelargonidin-3-O-arabinoside	Pelargonidin	up
Anthocyanidin_67	Pelargonidin-3-O-galactoside	Pelargonidin	up
Anthocyanidin_78	Peonidin-3,5-O-diglucoside	Peonidin	up
Anthocyanidin_85	Peonidin-3-O-galactoside	Peonidin	up
Anthocyanidin_86	Peonidin-3-O-glucoside	Peonidin	up
Anthocyanidin_88	Peonidin-3-O-rutinoside	Peonidin	up
Anthocyanidin_104	Procyanidin A2	Procyanidin	up
Anthocyanidin_105	Procyanidin B1	Procyanidin	up
Anthocyanidin_106	Procyanidin B2	Procyanidin	up
Anthocyanidin_107	Procyanidin B3	Procyanidin	up
Anthocyanidin_108	Procyanidin C1	Procyanidin	up
Anthocyanidin_116	Cyanidin-3-xylosyl-galactoside	Cyanidin	up
Anthocyanidin_117	Cyanidin-3-O-arabinosidase-glucos	Cyanidin	up

ID	Name	Class	Regulated
	ide		
Anthocyanidin_123	Cyanidin-3-gentiobioside	Cyanidin	up
Anthocyanidin_134	Cyanidin-3-malonyl-glucosyl-glucoside	Cyanidin	up
Anthocyanidin_145	Cyanidin-caffeoyl-glucoside-glucoside	Cyanidin	up
Anthocyanidin_208	Delphinidin-3-O-xyloside	Delphinidin	up
Anthocyanidin_214	Delphinidin-3-(6"-O-coumaroyl)galactoside	Delphinidin	up
Anthocyanidin_245	Malvidin-3-O-(6"-O-acetyl)glucoside	Malvidin	up
Anthocyanidin_277	Pelargonidin-3-O-(6"-O-xylosyl)galactoside	Pelargonidin	up
Anthocyanidin_278	Pelargonidin-3-O-rhamnoside-5-O-glucoside	Pelargonidin	up
Anthocyanidin_279	Pelargonidin-3-O-coumaroyl-5-O-galactoside	Pelargonidin	down
Anthocyanidin_319	Peonidin-3-O-xyloside	Peonidin	up
Anthocyanidin_401	Cyanidin-3,5-O-digalactoside	Cyanidin	up
Flavonoid_02	Miquelianin	Flavonols	up
Flavonoid_06	Diosmetin	Flavones	up
Flavonoid_16	7,4'-Di-O-methylapigenin	Flavones	up
Flavonoid_23	Quercetin	Flavonols	up
Flavonoid_40	Dihydromyricetin	Flavanonols	up
Flavonoid_50	Luteolin	Flavones	down
Flavonoid_54	Isorhamnetin-3-O-neohespeidoside	Flavonols	up
Flavonoid_55	(-)-Catechin	Flavanols	up
Flavonoid_57	Rutin	Flavonols	up
Flavonoid_61	Chrysin	Flavones	up
Flavonoid_65	Phlorizin	Chalcones	down
Flavonoid_74	Schaftoside	Flavone glycosides	up
Flavonoid_76	Sakuranetin	Flavanones	up
Flavonoid_79	Ligustroflavone	Phenonic acids	down
Flavonoid_86	Avicularin	Flavonols	down
Flavonoid_90	Cynaroside	Flavones	up
Flavonoid_96	Calycosin-7-O-β-D-glucoside	Isoflavanones	down
Flavonoid_99	Acacetin	Flavones	up
Flavonoid_108	Pinocembrin	Flavanones	up
Flavonoid_112	Echinatin	Chalcones	down
Flavonoid_119	Astragalin	Flavonols	down
Flavonoid_126	Eriodictyol	Flavanones	down
Flavonoid_137	Tiliroside	Flavonols	down

ID	Name	Class	Regulated
Flavonoid_138	Baimaside	Flavonols	down
Flavonoid_139	Dihydrokaempferol	Flavanonols	up
Flavonoid_146	Icariin	Flavonols	up
Flavonoid_154	Vitexin	Flavone glycosides	up
Flavonoid_157	Isomangiferin	Xanthones	down
Flavonoid_158	Isosakuranetin	Flavanones	up
Flavonoid_166	Genkwanin	Flavones	up
Flavonoid_172	Kaempferitrin	Flavonols	up
Flavonoid_176	Naringenin-7-glucoside	Flavanones	down
Flavonoid_177	Phloretin	Chalcones	up
Flavonoid_179	Poncirin	Flavanones	up
Flavonoid_181	Demethyltexasin	Isoflavanones	up
Flavonoid_194	Quercimeritrin	Flavonols	down
Flavonoid_201	Trilobatin	Chalcones	up
Flavonoid_202	Sieboldin	Chalcones	down
Flavonoid_204	Linarin	Flavones	up
Flavonoid_206	Salicylic acid	Benzoic acid and its derivatives	up
Flavonoid_207	4-Hydroxycinnamic acid	Phenylpropionic acids	down
Flavonoid_211	Resveratrol	Stilbenes	down
Flavonoid_217	Sophoraflavonoloside	Flavonols	up
Flavonoid_221	Eupafolin	Flavones	down
Flavonoid_227	2,6-Dihydroxybenzoic acid	Benzoic acid and its derivatives	up
Flavonoid_233	Norartocarpetin	Flavones	down
Flavonoid_236	Sinapaldehyde	Aromatic aldehydes	up
Flavonoid_244	Chlorogenic acid	Phenylpropionic acids	down
Flavonoid_245	Quercetin 3-O-neohesperidoside	Flavonols	up
Flavonoid_246	Rutin Trihydrate	Flavonols	up
Flavonoid_248	Aesculin	Coumarins	down
Flavonoid_249	Protocatechuic acid	Benzoic acid and its derivatives	up
Flavonoid_257	Gallic acid	Benzoic acid and its derivatives	down
Flavonoid_259	Isohemiphloin	Flavanones	down
Flavonoid_262	4-Hydroxybenzoic acid	Benzoic acid and its derivatives	down
Flavonoid_263	Cryptochlorogenic acid	Phenylpropionic acids	down

ID	Name	Class	Regulated
Flavonoid_264	Trifolin	Flavonols	down
Flavonoid_267	Morin	Flavonols	up
Flavonoid_271	Quercetin 3-sambubioside	Flavonols	down
Flavonoid_272	Chrysoeriol	Flavones	up
Flavonoid_276	3-O-Acetylpinobanksin	Flavanonols	up
Flavonoid_281	Hesperetin 7-O-glucoside	Flavanones	down
Flavonoid_283	Salicin	Glycosides	up
Flavonoid_296	kaempferol 7-O-glucoside	Flavonols	down
Flavonoid_307	Coniferaldehyde	Phenonic acids	up
Flavonoid_309	4',7-Di-O-methylnaringenin	Flavanones	up
Flavonoid_312	Isorhamnetin 3,7-O-diglucoside	Flavonols	down
Flavonoid_326	Acetovanillone	Phenonic acids	down
Flavonoid_328	2,4-Dihydroxybenzoic acid	Benzoic acid and its derivatives	up
Flavonoid_338	Apiin	Flavones	down
Flavonoid_343	Didymin	Flavanones	up
